# Combination of Drug Delivery Properties of PAMAM Dendrimers and Cytotoxicity of Platinum(IV) Complexes—A More Selective Anticancer Treatment?

**DOI:** 10.3390/pharmaceutics15051515

**Published:** 2023-05-17

**Authors:** Yvonne Lerchbammer-Kreith, Michaela Hejl, Petra Vician, Michael A. Jakupec, Walter Berger, Mathea S. Galanski, Bernhard K. Keppler

**Affiliations:** 1 Institute of Inorganic Chemistry, Faculty of Chemistry, University of Vienna, Waehringer Strasse 42, 1090 Vienna, Austria; 2 Center for Cancer Research and Comprehensive Cancer Center, Medical University of Vienna, Borschkegasse 8a, 1090 Vienna, Austria; 3 Research Cluster “Translational Cancer Therapy Research”, University of Vienna, Waehringer Strasse 42, 1090 Vienna, Austria

**Keywords:** platinum(IV) complexes, PAMAM dendrimers, anticancer, drug delivery

## Abstract

Based on their drug delivery properties and activity against tumors, we combined PAMAM dendrimers with various platinum(IV) complexes in order to provide an improved approach of anticancer treatment. Platinum(IV) complexes were linked to terminal NH_2_ moieties of PAMAM dendrimers of generation 2 (G2) and 4 (G4) via amide bonds. Conjugates were characterized by ^1^H and ^195^Pt NMR spectroscopy, ICP-MS and in representative cases by pseudo-2D diffusion-ordered NMR spectroscopy. Additionally, the reduction behavior of conjugates in comparison to corresponding platinum(IV) complexes was investigated, showing a faster reduction of conjugates. Cytotoxicity was evaluated via the MTT assay in human cell lines (A549, CH1/PA-1, SW480), revealing IC_50_ values in the low micromolar to high picomolar range. The synergistic combination of PAMAM dendrimers and platinum(IV) complexes resulted in up to 200 times increased cytotoxic activity of conjugates in consideration of the loaded platinum(IV) units compared to their platinum(IV) counterparts. The lowest IC_50_ value of 780 ± 260 pM in the CH1/PA-1 cancer cell line was detected for an oxaliplatin-based G4 PAMAM dendrimer conjugate. Finally, in vivo experiments of a cisplatin-based G4 PAMAM dendrimer conjugate were performed based on the best toxicological profile. A maximum tumor growth inhibition effect of 65.6% compared to 47.6% for cisplatin was observed as well as a trend of prolonged animal survival.

## 1. Introduction

Five decades after the discovery of the anticancer activity of cisplatin, platinum(II)-based anticancer agents still play an essential role in modern cancer treatment. The breakthrough of platinum(II) complexes was further consolidated by the introduction of carboplatin and oxaliplatin in clinical practice [1,2,3]. Together, these three platinum(II) drugs are integrated into about 50% of all cancer chemotherapies worldwide and cisplatin belongs to the most lucrative anticancer agents [4,5]. Despite the enormous success, platinum(II)-based cancer treatment lacks selectivity against tumor cells, leading to severe side effects, and is further affected by intrinsic and/or acquired resistance, diminishing its clinical efficacy [6].

The prodrug strategy, employing kinetically more inert platinum(IV) complexes, enabled a new approach to improve selectivity and reduce systemic toxicity. The reduction of the corresponding platinum(II) complexes, required to unleash their cytotoxic capacities, is facilitated by the characteristic oxygen-deficient milieu of tumor tissue [7,8]. Despite intensive research and promising (pre)clinical studies, platinum(IV) complexes such as tetraplatin, iproplatin, satraplatin and LA-12 could not show an overall improvement so far compared to platinum(II)-based anticancer agents [9,10,11].

Consequently, in order to improve selectivity, tumor-targeting strategies have attracted more and more attention. Passive tumor targeting exploits the enhanced permeability and retention (EPR) effect, a characteristic of cancerous tissue. The gaps formed between endothelial cells during tumor angiogenesis enable the infiltration of nanoparticles. Additionally, insufficient lymph drainage leads to an enhanced accumulation of macromolecules. Therefore, the development of drug delivery molecules in the nanometer range is of special interest [12,13].

Promising candidates can be found within the class of dendrimers, symmetrically designed polymeric nanostructures with adjustable functionalities such as size, shape and surface groups. The most intensively investigated representative is the poly(amidoamine) (PAMAM) dendrimer, developed by Donald Tomalia in 1985 [14,15]. Sequences of amidoamine units are constituted radially around an ethylenediamine core and the dendritic structure grows symmetrically controlled, using a two-step mechanism. Each full layer thereby represents a full generation, with primary amines serving as terminal groups. The surface functionalities enable covalent bonding or coordination of drugs, whereas small bioactive molecules can be encapsulated in the interior [16,17,18].

The beneficial combination of platinum complexes and PAMAM dendrimers was already shown in previous studies. Encapsulation of cisplatin led to higher anticancer potency and improved cellular accumulation even in cisplatin-resistant cell lines [19,20]. Additionally, efficacy and biodistribution studies in mice xenografts with ovarian cancer showed an increase in lifespan by up to 40%. Furthermore, the enhanced plasma concentration and tissue accumulation could allow a lower dosage, resulting in decreased side effects [21].

Besides encapsulation, attachment of cisplatin and doxorubicin to the surface of PAMAM dendrimers enabled a more efficacious treatment of breast cancer in animal experiments. Due to the improved accumulation of the conjugate in cancerous tissue, further tumor growth was prevented without noticeable adverse effects [22].

Previously, our group reported on significantly increased cytotoxicity achieved by the formation of amide bonds between oxaliplatin-based platinum(IV) complexes and full-generation PAMAM dendrimers [23].

Hence, we continue investigations into the potential of platinum(IV) compounds in combination with PAMAM dendrimers as anticancer agents in the present work. In total, 24 conjugates with various platinum(IV) complexes and PAMAM dendrimers of generation 2 (G2) and 4 (G4) were synthesized and characterized by multinuclear NMR spectroscopy and inductively coupled plasma mass spectrometry (ICP-MS). Cytotoxicity of the conjugates was compared to that of the corresponding platinum(IV) complexes and unloaded PAMAM dendrimers via the MTT assay in three human cancer cell lines. Finally, biodistribution and anticancer activity were investigated in a murine CT26 solid tumor model.

## 2. Materials and Methods

### 2.1. Materials

All chemicals and solvents were purchased from commercial suppliers and were used without further purification. K_2_[PtCl_4_] was acquired from Johnson Matthey (assay: 46.69% Pt) (Zurich, Switzerland), whereas G2 (20 wt.% in methanol) and G4 PAMAM dendrimers (10 wt.% in methanol) were purchased from Sigma-Aldrich (St. Louis, MO, USA). Additionally, the following chemicals were used as received: succinic anhydride (≥99%) (Sigma-Aldrich, Steinheim, Germany), absolute DMF (99.8%, extra dry, water <50 ppm) (Acros Organics, Geel, Belgium), N-(3-dimethylaminopropyl)-N′-ethylcarbodiimide hydrochloride (EDC·HCl) (>98%) (TCI Europe, Zwijndrecht, Belgium), N-hydroxysuccinimide (NHS) (98%) (Sigma-Aldrich, Steinheim, Germany).

Trial Kit Spectra/Por^®^ 7 (MWCO 1.0 kDa) and Spectra/Por^®^ 3 (MWCO 3.5 kDa) dialysis tubings were supplied by Carl Roth (Karlsruhe, Germany). Milli-Q water (18.2 MΩ cm, Milli-Q Advantage) was used for aqueous solutions as well as for preparative RP-HPLC purifications. All reactions involving platinum complexes were performed under light protection and with glass-coated magnetic stirring bars.

### 2.2. Preparative RP-HPLC

An Agilent 1200 Series system controlled by ChemStation^®^ software was used for purifications by preparative RP-HPLC. A XBridge^®^ Prep C18 10 µm OBD^TM^ Column (19 mm × 250 mm) from Waters served as the stationary phase, whereas different ratios of Milli-Q water, acetonitrile and methanol with the addition of 0.1% formic acid were used as eluents.

### 2.3. Elemental Analysis

The Microanalytical Laboratory of the Faculty of Chemistry at the University of Vienna performed the elemental analyses of the platinum(IV) compounds, using an Eurovector EA3000 elemental analyzer. All obtained values are in the range of ±0.4% of the calculated values, therefore confirming purity of at least 96%.

### 2.4. NMR Spectroscopy

NMR spectra were recorded on a Bruker AVANCE NEO 500 MHz spectrometer at 500.32 (^1^H), 125.81 (^13^C), 50.70 (^15^N), 470.56 (^19^F), and 107.38 MHz (^195^Pt) or an AVANCE III HD 700 MHz spectrometer at 659.03 MHz (^19^F) at 25 °C. The solvent resonances were used as internal references for ^1^H (d_6_-DMSO, δ = 2.50 ppm; d_7_-DMF, δ = 2.75 ppm (high field signal); D_2_O, δ = 4.79 ppm) and ^13^C (d_6_-DMSO, δ = 39.51 ppm; d_7_-DMF, δ = 29.76 ppm (high field signal)), ^19^F chemical shifts are given relative to CCl_3_F, whereas NH_4_Cl and K_2_[PtCl_4_] served as external reference for ^15^N and ^195^Pt NMR spectroscopy.

DOSY experiments were measured with a standard Bruker DOSY pulse sequence without sample spinning in D_2_O at 298 K. The diffusion coefficient *D*, obtained from the spectra, was used for the estimation of the diameter of PAMAM dendrimers and conjugates by assuming a spherical size and using the Stokes–Einstein equation:

$$R_{0}=\frac{k_{B}T}{6\pi \eta D}$$

*R*_0_ is the (hydrodynamic) radius, *k_B_* the Boltzmann constant (JK^−1^), *T* the temperature [K], *η* the viscosity of the liquid (1.095 × 10^−3^ Pa·s) and *D* the diffusion coefficient [m^2^s^−1^] [23].

### 2.5. Reduction Behavior

The reduction of platinum(IV) complexes and representatives of each series of conjugates by ascorbic acid was observed by ^1^H NMR spectroscopic measurements at ambient temperature. An amount of 1 mM solutions of platinum(IV) complexes **1**–**3**, **5**–**7** as well as of the conjugates **C2**, **C11**, **C13**, **C22**–**C24** referred to their corresponding platinum units were prepared in a D_2_O phosphate-buffered solution (50 mM) at physiological pD = 7.4. Afterwards, ascorbic acid was added as a reducing agent (25 mM, 25 eq.) and ^1^H NMR measurements were performed for several days. The reduction behavior for substances **1**–**3**, **5**, **6**, **C2**, **C11**, **C13**, **C22**, **C23** was monitored by the resulting decrease in intensity of the acetato signal (release of acetic acid). The percentage of reduced species was determined by using the integration ratios of these two signals. Contrarily, ratios of signals of the succinato ligands and free succinic acid were consulted for the reduction determination of substances **7** and **C24**. However, the reduction half-time of **C24** could not be obtained due to the superimposition of the succinato peaks with other signals.

### 2.6. ICP-MS

Digestion of all conjugates (0.5–1.5 mg) was performed in 2 mL of HNO_3_ (20%) and 0.1 mL of H_2_O_2_ (30%) with a temperature-controlled heating plate of graphite from Labter. After dilution (1:10,000) of digested samples with HNO_3_ (3%), the total platinum amount was determined with an Agilent 7800 ICP-MS instrument. For each sample, 10 measurements were performed and the obtained data were analyzed with the Agilent MassHunter software package (Workstation Software, Version C.01.04, 2018, Agilent, Santa Clara, CA, USA).

### 2.7. Cytotoxicity Tests

Culture of and 96 h cytotoxicity tests in the human cell lines CH1/PA-1 (ovarian teratocarcinoma), SW480 (colon carcinoma) and A549 (non-small cell lung cancer) were performed as described previously [24], with the exception that test compounds were dissolved either in sterile water or supplemented MEM and then serially diluted in the latter medium.

The MTT assay in the murine cell line CT26 was performed as described in [25].

### 2.8. In Vivo Experiments and Organ Distribution

Female and male BALB/c mice, bread in-house (originally Janvier), were kept in a controlled environment under pathogen-free conditions. By the toxicity tests, non-tumor-bearing female BALB/c mice were treated intravenously at a dose: G4 PAMAM dendrimer at 0.058 mg/20 g, **C11** at 0.17 mg/20 g, **C12** at 0.075 mg/20 g and 0.15 mg/20 g, **C13** at 0.91 mg/20 g and **C22** at 0.52 mg/20 g three times in the first week. All drugs were dissolved in 0.9% NaCl. For the efficacy study, CT26 (5 × 10^5^ in 50 μL serum-free RMPI medium) murine colon cells were injected into the right flank (subcutaneously) of female BALB/c mice. When all tumors were measurable (at day 5), animals were treated intravenously with solvent (0.9% NaCl, 100 μL/20 g), **C12** (7.5 mg/kg in 0.9% NaCl, 100 μL/20 g) and cisplatin (3 mg/kg in 0.9% NaCl, 100 μL/20 g) twice a week for two weeks. Tumor volume and size (measured by caliper) and body weight were evaluated every working day; the animals were sacrificed by cervical dislocation upon a loss in body weight, tumor size or other indications of deteriorated health. In vivo experiments were done according to the regulations of the Ethics Committee for the Care and Use of Laboratory Animals at the Medical University Vienna (proposal number BMBWF-V/3b 2020-0.380.502).

Organs and tumors for platinum content/distribution were harvested 24 h after the second application of **C12** (7.5 mg/kg in 0.9% NaCl, 100 μL/20 g) and cisplatin (3 mg/kg in 0.9% NaCl, 100 μL/20 g) in male CT26-allograft-bearing BALB/c mice. Blood was taken after 30 min and 24 h after the first application from the facial vein and terminally by cardiac puncture 24 h after the second application. To separate serum from blood pellet, blood was twice spun down at 900 g for 10 min at 4 °C. The platinum amount of all samples was measured by ICP-MS.

### 2.9. Synthesis

#### 2.9.1. Platinum(IV) Complexes

Acetatohydroxidoplatinum(IV) complexes serving as precursors for substances **1**–**6** as well as substance **7** were synthesized according to previously published procedures [23,26,27,28].

General procedure 1: carboxylation of unsymmetrically oxidized platinum(IV) complexes (**1**–**6**):

The corresponding precursor platinum(IV) complex and succinic anhydride were stirred overnight in absolute DMF under argon atmosphere at 50 °C. The solvent was removed under reduced pressure. Purification was performed by using preparative RP-HPLC. Finally, the product was freeze-dried.

1. (OC-6-44)-Acetatodiammine(3-carboxypropanoato)dichloridoplatinum(IV) (**1**)

The reaction was performed according to general procedure 1. Acetatodiamminedichloridohydroxidoplatinum(IV) (5.171 g, impure, 13.75 mmol, 1 eq.), succinic anhydride (5.502 g, 55.00 mmol, 4 eq.), absolute DMF (100 mL). Preparative RP-HPLC (isocratic, MeOH: Milli-Q water = 5:95 + 0.1% formic acid). Yield: 1.555 g. ^1^H NMR (d_6_-DMSO): δ = 6.51 (b, 6H, NH_3_), one of the CH_2_ signals of the succinato ligand is in part overlapping with the DMSO solvent peak, 2.34–2.38 (m, 2H, CH_2_, succinato), 1.90 (s, 3H, CH_3_) ppm. Elemental analysis: C_6_H_14_Cl_2_N_2_O_6_Pt∙H_2_O; calcd. C 14.58, H 3.26, N 5.67, found C 14.40, H 3.14, N 5.64.

2. (OC-6-44)-Acetaodiammine(3-carboxypropanoato)(cyclobutane-1,1-dicarboxylato)platinum(IV) (**2**)

The reaction was performed according to general procedure 1. Acetaodiammine(cyclobutane-1,1-dicarboxylato)hydroxidoplatinum(IV) (2.260 g, impure, 5.05 mmol, 1 eq.), succinic anhydride (2.021 g, 20.21 mmol, 4 eq.), absolute DMF (40 mL). Preparative RP-HPLC (isocratic, MeOH: Milli-Q water = 17:83 + 0.1% formic acid). Yield: 524 mg. ^1^H NMR (d_6_-DMSO): δ = 6.34 (t, ^1^J(^14^N, ^1^H) = 50.5 Hz, 6H, NH_3_), CH_2_-C signal of the cyclobutyl moiety and one of the CH_2_ signals of the succinato ligand are in part overlapping with the DMSO solvent peak, 2.35 (m, 2H, CH_2_, succinato), 1.89 (s, 3H, CH_3_), 1.80 (p, ^3^J(^1^H, ^1^H = 8.2 Hz), 2H, CH_2_, cyclobutyl) ppm. Elemental analysis: C_12_H_20_N_2_O_10_Pt∙H_2_O; calcd. C 25.49, H 3.92, N 4.95, found C 25.80, H 3.66, N 5.12.

3. (OC-6-44)-Acetato(3-carboxypropanoato)(1R,2R-cyclohexane-1,2-diamine)oxalatoplatinum(IV) (**3**)

The reaction was performed according to general procedure 1. Acetato(1R,2R-cyclohexane-1,2-diamine)hydroxidooxalatoplatinum(IV) (7.845 g, impure, 16.64 mmol, 1 eq.), succinic anhydride (6.666 g, 66.71 mmol, 4 eq.), absolute DMF (130 mL). Preparative RP-HPLC (isocratic, ACN: Milli-Q water = 5:95 + 0.1% formic acid). Yield: 2.988 g. Crystals suitable for X-ray diffraction were obtained from a methanol solution by slow evaporation at room temperature. ^1^H NMR (d_6_-DMSO): δ = 12.13 (b, 1H, OH), 8.29 (m, 4H, NH_2_), 2.53–2.62 (m, 2H, CH, DACH), one of the CH_2_ signals of the succinato ligand is overlapping with the DMSO solvent peak, 2.35–2.41 (m, 2H, CH_2_, succinato), 2.06–2.14 (m, 2H, CH_2_, DACH), 1.95 (s, 3H, CH_3_), 1.47–1.54 (m, 2H, CH_2_, DACH), 1.33–1.47 (m, 2H, CH_2_, DACH) 1.08–1.21 (m, 2H, CH_2_, DACH) ppm. Elemental analysis: C_14_H_22_N_2_O_10_Pt∙H_2_O; calcd. C 28.43, H 4.09, N 4.74, found C 28.08, H 4.00, N 4.91.

4. (OC-6-44)-Acetato(3-carboxypropanoato)(1R,2R-cyclohexane-1,2-diamine)(^13^C_2_)oxalatoplatinum(IV) (**4**)

The reaction was performed according to general procedure 1. Acetato(1R,2R-cyclohexane-1,2-diamine)(^13^C_2_)hydroxidooxalatoplatinum(IV) (677 mg, impure, 1.42 mmol, 1eq.), succinic anhydride (570 mg, 5.70 mmol, 4 eq.), absolute DMF (30 mL). Preparative RP-HPLC (isocratic, ACN: Milli-Q water = 5:95 + 0.1% formic acid). Yield: 188 mg. ^1^H NMR (D_2_O): δ = 2.83–2.95 (m, 2H, DACH), 2.64–2.69 (m, 2H, succinato), 2.59–2.63 (m, 2H, succinato), 2.27–2.34 (m, 2H, DACH), 2.07 (s, 3H, CH_3_), 1.53–1.70 (m, 4H, DACH), 1.20–1.32 (m, 2H, DACH) ppm. ^13^C NMR (D_2_O): δ = 181.9 (C=O, succinato), 181.4 (C=O, acetato), 177.2 (C=O, succinato), 166.4 (^13^C=O, oxalato), 164.5–165.5 (smaller ^13^C=O signals, impurities), 61.8 (CH, DACH), 61.3 (CH, DACH), 30.8 (CH_2_, DACH), 30.7 (CH_2_, succinato), 29.7 (CH_2_, succinato), 23.42 (CH_2_, DACH), 23.38 (CH_2_, DACH), 22.1 (CH_3_) ppm. ^195^Pt NMR (D_2_O): δ = 3214 ppm. Elemental analysis: C_12_^13^C_2_H_22_N_2_O_10_Pt∙1.5H_2_O; calcd. C 28.00, H 4.20, N 4.67, found C 27.91, H 3.82, N 4.89.

5. (OC-6-54)-Acetato(3-carboxypropanoato)(^13^C_2_)oxalato(1R,2R,4R/1S,2S,4S)-(4-trifluoromethyl-cyclohexane-1,2-diamine)platinum(IV) (**5**)

The reaction was performed according to general procedure 1. Acetatohydroxido(^13^C_2_)oxalato(1R,2R,4R/1S,2S,4S)-(4-trifluoromethyl-cyclohexane-1,2-diamine)platinum(IV) (2.588 g, impure, 4.76 mmol, 1 eq.), succinic anhydride (1.192 g, 11.91 mmol, 2.5 eq.), absolute DMF (40 mL). Preparative RP-HPLC (isocratic, MeOH: Milli-Q water = 15:85 + 0.1% formic acid). Yield: 113 mg. ^1^H NMR (d_6_-DMSO): δ = 12.11 (b, 1H, OH), 8.27–8.57 (m, 2H, NH_2_), 7.96–8.27 (m, 2H, NH_2_), 2.81 (m, 1H, CH, DACH), 2.73 (m, 1H, CH, DACH), one CH_2_ signal of the succinato ligand and one CH signal of the DACH ligand is overlapping with the DMSO solvent peak, 2.34–2.43 (m, 2H, CH_2_, succinato), 2.20–2.28 (m, 1H, CH_2_, DACH), 2.13–2.20 (m, 1H, CH_2_, DACH), 1.96+1.95 (b, 3H, CH_3_), 1.71–1.78 (m, 1H, CH_2_, DACH), 1.48–1.63 (m, 2H, CH_2_, DACH), 1.28–1.40 (m, 1H, CH_2_, DACH) ppm. ^13^C NMR (d_6_-DMSO): δ = 179.5 + 179.2 (C=O, succinato), 178.4 + 178.2 (C=O, acetato), 173.85 + 173.75 (C=O, succinato), 163.34 + 163.32 (^13^C=O, oxalato), 162.7–165.8 (smaller ^13^C=O (coupling) signals based on the asymmetry of labeled oxalate), 126.9 (q, ^1^J (^19^F, ^13^C) = 278.6 Hz, CF_3_), 59.9 (CH, DACH), 59.3 (CH, DACH), 59.2 (CH, DACH), 30.64 + 30.58 (CH_2_, succinato), 29.8 + 29.7 (CH_2_, succinato), 29.1 + 29.0 (CH_2_, DACH), 28.1 + 28.0 (CH_2_, DACH), 23.0 + 22.9 (CH_3_), 22.48+22.45 (CH_2_, DACH) ppm. ^15^N NMR (d_6_-DMSO): δ = −8.4 (NH_2_) ppm. ^19^F NMR (d_6_-DMSO): δ = −71.37/−71.39 (2d, ^3^J(^1^H, ^19^F) = 8.6 Hz, 3F, CF_3_) ppm. ^195^Pt NMR (d_6_-DMSO): δ = 3260 (major), 3234 (minor) ppm. Elemental analysis: C_13_^13^C_2_H_21_F_3_N_2_O_10_Pt∙2H_2_O; calcd. C 26.59, H 3.73, N 4.14, found C 26.58, H 3.42, N 4.31.

6. (OC-6-44)-Acetato(3-carboxypropanoato)dichlorido(1R,2R-cyclohexane-1,2-diamine)platinum(IV) (**6**)

The reaction was performed according to general procedure 1. Acetatodichlorido(1R,2R-cyclohexane-1,2-diamine)hydroxidoplatinum(IV) (147 mg, 0.32 mmol, 1 eq.), succinic anhydride (82 mg, 0.81 mmol, 2.5 eq.), absolute DMF (15 mL). Preparative RP-HPLC (isocratic, ACN: Milli-Q water = 10:90 + 0.1% formic acid). Yield: 40 mg (22%). ^1^H NMR (d_6_-DMSO): δ = 12.10 (s, 1H, OH), 9.33 (m, 2H, NH_2_), 8.30 (b, 1H, NH_2_), 8.12 (b, 1H, NH_2_), 2.52–2.68 (m, 2H, CH, DACH), 2.33–2.49 (m, 4H, CH_2_, succinato signal is in part overlapping with the DMSO solvent peak), 2.14–2.23 (m, 2H, CH_2_, DACH), 1.95 (s, 3H, CH_3_), 1.46–1.56 (m, 2H, CH_2_, DACH), 1.21–1.39 (m, 2H, CH_2_, DACH), 1.08–1.20 (m, 2H, CH_2_, DACH) ppm. ^13^C NMR (d_6_-DMSO): δ = 181.9 (C=O, succinato), 180.7 (C=O, acetato), 173.7 (C=O, succinato), 62.45 + 62.43 (CH, DACH), 31.2 (CH_2_, succinato), 31.0 (CH_2_, DACH), 29.6 (CH_2_, succinato), 23.53 (CH_3_), 23.48 (CH_2_, DACH), 23.3 (CH_2_, DACH) ppm. ^15^N NMR (d_6_-DMSO): δ = 5.3 (NH_2_), 3.7 (NH_2_) ppm. ^195^Pt NMR (d_6_-DMSO): δ = 2729 ppm. Elemental analysis: C_12_H_22_Cl_2_N_2_O_6_Pt; calcd. C 25.91, H 3.99, N 5.04, found C 25.53, H 3.92, N 5.01.

7. (OC-6-44)-(3-carboxypropanoato)(1R,2R-cyclohexanem-1,2-diamine)hydroxidooxalatoplatinum(IV) (**7**)

(1R,2R-cyclohexanediamine)dihydroxidooxalatoplatinum(IV) (57 mg, 0.13 mmol, 1 eq.) and succinic anhydride (13 mg, 0.13 mmol, 1 eq.) were stirred in absolute DMSO (10 mL) for 16 h under argon atmosphere at 50 °C. Afterward, the solvent was removed under reduced pressure. Purification was performed via preparative RP-HPLC (isocratic, MeOH: Milli-Q water = 5:95 + 0.1% formic acid) and the final product was obtained after lyophilization. Yield: 25 mg (36%). ^1^H NMR (d_6_-DMSO): δ = 12.04 (b, 2H, OH), 8.42 (b, 1H, NH_2_), 8.09 (b, 1H, NH_2_), 7.80 (b, 1H, NH_2_), 7.06 (b, 1H, NH_2_), the CH signals of the DACH ligand are overlapping with the DMSO solvent peak, 2.40–2.45 (m, 2H, CH_2_, succinato), 2.34–2.39 (m, 2H, CH_2_, succinato), 2.00–2.11 (m, 2H, CH_2_, DACH), 1.39–1.55 (m, 3H, CH_2_, DACH), 1.26–1.36 (m, 1H, CH_2_, DACH), 1.05–1.18 (m, 2H, CH_2_, DACH) ppm. ^13^C NMR (d_6_-DMSO): δ = 180.8 (C=O, succinato), 173.9 (C=O, succinato), 163.9 (C=O, oxalato), 163.8 (C=O, oxalato), 61.2 (CH, DACH), 60.0 (CH, DACH), 31.4 (CH_2_, succinato), 30.9 (CH_2_, DACH), 30.7 (CH_2_, DACH), 29.8 (CH_2_, succinato), 23.7 (CH_2_, DACH), 23.5 (CH_2_, DACH) ppm. ^15^N NMR (d_6_-DMSO): δ = −5.9 (NH_2_) and −9.6 (NH_2_) ppm. ^195^Pt NMR (d_6_-DMSO): δ = 3039 ppm. Elemental analysis: C_12_H_20_N_2_O_9_Pt∙2H_2_O; calcd. C 25.40, H 4.25, N 4.94, found C 25.74, H 3.85, N 5.02.

#### 2.9.2. Platinum(IV)-PAMAM Dendrimer Conjugates

##### General Procedure 2: Coupling of Platinum(IV) Complexes to G2 or G4 PAMAM Dendrimers (**C1**–**C27**)

The corresponding platinum(IV) complex **1**–**7** (1 eq.), N-(3-dimethylaminopropyl)-N′-ethylcarbodiimide hydrochloride (EDC·HCl) (3.5 eq.) and N-hydroxysuccinimide (NHS) (3.5 eq.) were dissolved in Milli-Q water (5 mL) and the mixture was stirred for about 40 min at room temperature. Afterward, an aqueous solution (15 mL) of PAMAM dendrimer (0.06 eq. for G2 and 0.02 eq. for G4) was added. The reaction time ranged from 6 to 24 h. Purification was performed by dialysis against distilled water (MWCO = 1.0 kDa for G2 and 3.5 kDa for G4, 12 changes within 14 h). Finally, the product was obtained via lyophilization.

1. Complex **1** coupled to G2 PAMAM dendrimer (**C1**)

The reaction was performed according to general procedure 2. **1** (60 mg, 0.13 mmol, 1 eq.), G2 PAMAM (25 mg, 0.0076 mmol, 0.06 eq.), EDC·HCl (85 mg, 0.44 mmol, 3.5 eq.), NHS (51 mg, 0.44 mmol, 3.5 eq.), reaction time of 12 h. Yield: 29 mg. ICP-MS (Pt): 194.1 g/kg, average Pt loading of 37.31%, 5.97 Pt units. ^195^Pt NMR (D_2_O): δ = 2712 ppm.

2. Complex **2** coupled to G2 PAMAM dendrimer (**C2**)

The reaction was performed according to general procedure 2. **2** (60 mg, 0.11 mmol, 1 eq.), G2 PAMAM (21 mg, 0.0066 mmol, 0.06 eq.), EDC·HCl (74 mg, 0.38 mmol, 3.5 eq.), NHS (44 mg, 0.38 mmol, 3.5 eq.), reaction time of 12 h. Yield: 44 mg. ICP-MS (Pt): 161.7 g/kg, average Pt loading of 30.13%, 4.82 Pt units. ^195^Pt NMR (D_2_O): δ = 3507 ppm.

3. Complex **4** coupled to G2 PAMAM dendrimer (**C3**)

The reaction was performed according to general procedure 2. **4** (60 mg, 0.10 mmol, 1 eq.), G2 PAMAM (20 mg, 0.0063 mmol, 0.06 eq.), EDC·HCl (70 mg, 0.37 mmol, 3.5 eq.), NHS (42 mg, 0.37 mmol, 3.5 eq.), reaction time of 12 h. Yield: 23 mg. ICP-MS (Pt): 176.2 g/kg, average Pt loading of 37.06%, 5.93 Pt units. ^195^Pt NMR (D_2_O): δ = 3218 ppm.

4. Complex **5** coupled to G2 PAMAM dendrimer (**C4**)

The reaction was performed according to general procedure 2. **5** (151 mg, 0.23 mmol, 1 eq.), G2 PAMAM (46 mg, 0.014 mmol, 0.06 eq.), EDC·HCl (157 mg, 0.82 mmol, 3.5 eq.), NHS (94 mg, 0.82 mmol, 3.5 eq.), reaction time of 12h. Yield: 148 mg. ICP-MS (Pt): 140.7 g/kg, average Pt loading of 27.38%, 4.38 Pt units. ^19^F NMR (D_2_O): δ = −72.8–(−72.5) (d, ^3^J(^1^H, ^19^F) = 8.6 Hz, 3F, C*F*_3_) (several overlapping duplets) ppm. ^195^Pt NMR (D_2_O): δ = 3246 (minor), 3239 (major) ppm.

5. Complex **7** coupled to G2 PAMAM dendrimer (**C5**)

The reaction was performed according to general procedure 2. **7** (80 mg, 0.15 mmol, 1 eq.), G2 PAMAM (29 mg, 0.009 mmol, 0.06 eq.), EDC·HCl (101 mg, 0.53 mmol, 3.5 eq.), NHS (61 mg, 0.53 mmol, 3.5 eq.), reaction time of 12 h. Yield: 45 mg. ICP-MS (Pt): 207.7 g/kg, average Pt loading of 47.88%, 7.66 Pt units. ^195^Pt NMR (D_2_O): δ = 3038 ppm.

6. Complex **1** coupled to G4 PAMAM dendrimer (**C6**)

The reaction was performed according to general procedure 2. **1** (60 mg, 0.13 mmol, 1 eq.), G4 PAMAM (36 mg, 0.0025 mmol, 0.02 eq.), EDC·HCl (85 mg, 0.44 mmol, 3.5 eq.), NHS (51 mg, 0.44 mmol, 3.5 eq.), reaction time of 12 h. Yield: 41 mg. ICP-MS (Pt): 93.2 g/kg, average Pt loading of 13.59%, 8.70 Pt units. ^195^Pt NMR (D_2_O): δ = 2711 ppm.

7. Complex **1** coupled to G4 PAMAM dendrimer (**C7**)

The reaction was performed according to general procedure 2. **1** (60 mg, 0.13 mmol, 1 eq.), G4 PAMAM (36 mg, 0.0025 mmol, 0.02 eq.), EDC·HCl (85 mg, 0.44 mmol, 3.5 eq.), NHS (51 mg, 0.44 mmol, 3.5 eq.), reaction time of 24 h. Yield: 26 mg. ICP-MS (Pt): 102.7 g/kg, average Pt loading of 15.42%, 9.87 Pt units. ^195^Pt NMR (D_2_O): δ = 2712 ppm.

8. Complex **1** coupled to G4 PAMAM dendrimer (**C8**)

The reaction was performed according to general procedure 2. **1** (60 mg, 0.13 mmol, 1 eq.), G4 PAMAM (36 mg, 0.0025 mmol, 0.02 eq.), EDC·HCl (85 mg, 0.44 mmol, 3.5 eq.), NHS (51 mg, 0.44 mmol, 3.5 eq.), reaction time of 12 h. Yield: 34.8 mg. ICP-MS (Pt): 105.3 g/kg, average Pt loading of 15.94%, 10.20 Pt units. ^195^Pt NMR (D_2_O): δ = 2712 ppm.

9. Complex **1** coupled to G4 PAMAM dendrimer (**C9**)

The reaction was performed according to general procedure 2. **1** (60 mg, 0.13 mmol, 1 eq.), G4 PAMAM (36 mg, 0.0025 mmol, 0.02 eq.), EDC·HCl (85 mg, 0.44 mmol, 3.5 eq.), NHS (51 mg, 0.44 mmol, 3.5 eq.), reaction time of 18 h. Yield: 33 mg. ICP-MS (Pt): 111.6 g/kg, average Pt loading of 17.23%, 11.03 Pt units. ^195^Pt NMR (D_2_O): δ = 2712 ppm.

10. Complex **1** coupled to G4 PAMAM dendrimer (**C10**)

The reaction was performed according to general procedure 2. **1** (60 mg, 0.13 mmol, 1 eq.), G4 PAMAM (36 mg, 0.0025 mmol, 0.02 eq.), EDC·HCl (85 mg, 0.44 mmol, 3.5 eq.), NHS (51 mg, 0.44 mmol, 3.5 eq.), reaction time of 21 h. Yield: 28 mg. ICP-MS (Pt): 129.5 g/kg, average Pt loading of 21.20%, 13.57 Pt units. ^195^Pt NMR (D_2_O): δ = 2711 ppm.

11. Complex **1** coupled to G4 PAMAM dendrimer (**C11**)

The reaction was performed according to general procedure 2. **1** (60 mg, 0.13 mmol, 1 eq.), G4 PAMAM (36 mg, 0.0025 mmol, 0.02 eq.), EDC·HCl (85 mg, 0.44 mmol, 3.5 eq.), NHS (51 mg, 0.44 mmol, 3.5 eq.), reaction time of 12 h. Yield: 54 mg. ICP-MS (Pt): 235.8 g/kg, average Pt loading of 60.33%, 38.61 Pt units. ^195^Pt NMR (D_2_O): δ = 2712 ppm.

12. Complex **1** coupled to G4 PAMAM dendrimer (**C12**)

The reaction was performed according to general procedure 2. **1** (300 mg, 0.63 mmol, 1 eq.), G4 PAMAM (179 mg, 0.013 mmol, 0.02 eq.), EDC·HCl (423 mg, 2.21 mmol, 3.5 eq.), NHS (254 mg, 2.21 mmol, 3.5 eq.), reaction time of 12 h. Yield: 325 mg. ICP-MS (Pt): 259.6 g/kg, average Pt loading of 75.98%, 48.63 Pt units. ^195^Pt NMR (D_2_O): δ = 2710 ppm.

13. Complex **2** coupled to G4 PAMAM dendrimer (**C13**)

The reaction was performed according to general procedure 2. **2** (60 mg, 0.131 mmol, 1 eq.), G4 PAMAM (31 mg, 0.0022 mmol, 0.02 eq.), EDC·HCl (73 mg, 0.38 mmol, 3.5 eq.), NHS (44 mg, 0.38 mmol, 3.5 eq.), reaction time of 12 h. Yield: 67 mg. ICP-MS (Pt): 195.1 g/kg, average Pt loading of 47.30%, 30.27 Pt units. ^195^Pt NMR (D_2_O): δ = 3507 ppm.

14. Complex **3** coupled to G4 PAMAM dendrimer (**C14**)

The reaction was performed according to general procedure 2. **3** (60 mg, 0.10 mmol, 1 eq.), G4 PAMAM (33 mg, 0.0021 mmol, 0.02 eq.), EDC·HCl (70 mg, 0.37 mmol, 3.5 eq.), NHS (42 mg, 0.37 mmol, 3.5 eq.), reaction time of 18 h. Yield: 80 mg. ICP-MS (Pt): 86.8 g/kg, average Pt loading of 13.14%, 8.41 Pt units. ^195^Pt NMR (D_2_O): δ = 3217 ppm.

15. Complex **3** coupled to G4 PAMAM dendrimer (**C15**)

The reaction was performed according to general procedure 2. **3** (60 mg, 0.10 mmol, 1 eq.), G4 PAMAM (33 mg, 0.0021 mmol, 0.02 eq.), EDC·HCl (70 mg, 0.37 mmol, 3.5 eq.), NHS (42 mg, 0.37 mmol, 3.5 eq.), reaction time of 15 h. Yield: 56 mg. ICP-MS (Pt): 103.9 g/kg, average Pt loading of 16.81%, 10.76 Pt units. ^195^Pt NMR (D_2_O): δ = 3217 ppm.

16. Complex **3** coupled to G4 PAMAM dendrimer (**C16**)

The reaction was performed according to general procedure 2. **3** (60 mg, 0.10 mmol, 1 eq.), G4 PAMAM (33 mg, 0.0021 mmol, 0.02 eq.), EDC·HCl (70 mg, 0.37 mmol, 3.5 eq.), NHS (42 mg, 0.37 mmol, 3.5 eq.), reaction time of 12 h. Yield: 90 mg. ICP-MS (Pt): 113.2 g/kg, average Pt loading of 19.03%, 12.18 Pt units. ^195^Pt NMR (D_2_O): δ = 3218 ppm.

17. Complex **3** coupled to G4 PAMAM dendrimer (**C17**)

The reaction was performed according to general procedure 2. **3** (60 mg, 0.10 mmol, 1 eq.), G4 PAMAM (33 mg, 0.0021 mmol, 0.02 eq.), EDC·HCl (71 mg, 0.37 mmol, 3.5 eq.), NHS (42 mg, 0.37 mmol, 3.5 eq.), reaction time of 24 h. Yield: 80 mg. ICP-MS (Pt): 119.1 g/kg, average Pt loading of 20.53%, 13.14 Pt units. ^195^Pt NMR (D_2_O): δ = 3218 ppm.

18. Complex **3** coupled to G4 PAMAM dendrimer (**C18**)

The reaction was performed according to general procedure 2. **3** (60 mg, 0.10 mmol, 1 eq.), G4 PAMAM (33 mg, 0.0021 mmol, 0.02 eq.), EDC·HCl (71 mg, 0.37 mmol, 3.5 eq.), NHS (42 mg, 0.37 mmol, 3.5 eq.), reaction time of 6 h. Yield: 81 mg. ICP-MS (Pt): 141.4 g/kg, average Pt loading of 26.98%, 17.27 Pt units. ^195^Pt NMR (D_2_O): δ = 3217 ppm.

19. Complex **3** coupled to G4 PAMAM dendrimer (**C19**)

The reaction was performed according to general procedure 2. **3** (60 mg, 0.10 mmol, 1 eq.), G4 PAMAM (33 mg, 0.0021 mmol, 0.02 eq.), EDC·HCl (70 mg, 0.37 mmol, 3.5 eq.), NHS (42 mg, 0.37 mmol, 3.5 eq.), reaction time of 21 h. Yield: 81 mg. ICP-MS (Pt): 160.7 g/kg, average Pt loading of 33.78%, 21.62 Pt units. ^195^Pt NMR (D_2_O): δ = 3218 ppm.

20. Complex **3** coupled to G4 PAMAM dendrimer (**C20**)

The reaction was performed according to general procedure 2. **3** (60 mg, 0.10 mmol, 1 eq.), G4 PAMAM (33 mg, 0.0021 mmol, 0.02 eq.), EDC·HCl (71 mg, 0.37 mmol, 3.5 eq.), NHS (43 mg, 0.37 mmol, 3.5 eq.), reaction time of 9 h. Yield: 95 mg. ICP-MS (Pt): 181.4 g/kg, average Pt loading of 42.80%, 27.39 Pt units. ^195^Pt NMR (D_2_O): δ = 3218 ppm.

21. Complex **3** coupled to G4 PAMAM dendrimer (**C21**)

The reaction was performed according to general procedure 2. **3** (60 mg, 0.10 mmol, 1 eq.), G4 PAMAM (33 mg, 0.0021 mmol, 0.02 eq.), EDC·HCl (70 mg, 0.37 mmol, 3.5 eq.), NHS (42 mg, 0.37 mmol, 3.5 eq.), reaction time of 24 h. Yield: 76 mg. ICP-MS (Pt): 240.1 g/kg, average Pt loading of 86.73%, 55.51 Pt units. ^195^Pt NMR (D_2_O): δ = 3217 ppm.

22. Complex **4** coupled to G4 PAMAM dendrimer (**C22**)

The reaction was performed according to general procedure 2. **4** (60 mg, 0.10 mmol, 1 eq.), G4 PAMAM (30 mg, 0.0021 mmol, 0.02 eq.), EDC·HCl (70 mg, 0.37 mmol, 3.5 eq.), NHS (43 mg, 0.37 mmol, 3.5 eq.), reaction time of 12 h. Yield: 65 mg. ICP-MS (Pt): 168.1 g/kg, average Pt loading of 37.95%, 24.29 Pt units. ^195^Pt NMR (D_2_O): δ = 3218 ppm.

23. Complex **5** coupled to G4 PAMAM dendrimer (**C23**)

The reaction was performed according to general procedure 2. **5** (60 mg, 0.09 mmol, 1 eq.), G4 PAMAM (27 mg, 0.0019 mmol, 0.02 eq.), EDC·HCl (63 mg, 0.33 mmol, 3.5 eq.), NHS (38 mg, 0.33 mmol, 3.5 eq.), reaction time of 12 h. Yield: 57 mg. ICP-MS (Pt): 125.1 g/kg, average Pt loading of 23.80%, 15.23 Pt units. ^19^F NMR (D_2_O): δ = -72.9-(-72.4) (d, ^3^J (^1^H, ^19^F) = 8.5 Hz, 3F, C*F*_3_) (several overlapping duplets) ppm. ^195^Pt NMR (D_2_O): δ = 3246 (minor), 3239 (major) ppm.

24. Complex **7** coupled to G4 PAMAM dendrimer (**C24**)

The reaction was performed according to general procedure 2. **7** (80 mg, 0.15 mmol, 1 eq.), G4 PAMAM (43 mg, 0.0030 mmol, 0.02 eq.), EDC·HCl (101 mg, 0.53 mmol, 3.5 eq.), NHS (61 mg, 0.53 mmol, 3.5 eq.), reaction time of 12 h. Yield: 106 mg. ICP-MS (Pt): 152.6 g/kg, average Pt loading of 29.06%, 18.60 Pt units. ^195^Pt NMR (D_2_O): δ = 3038 ppm.

## 3. Results and Discussion

### 3.1. Synthesis

**Scheme 1:**
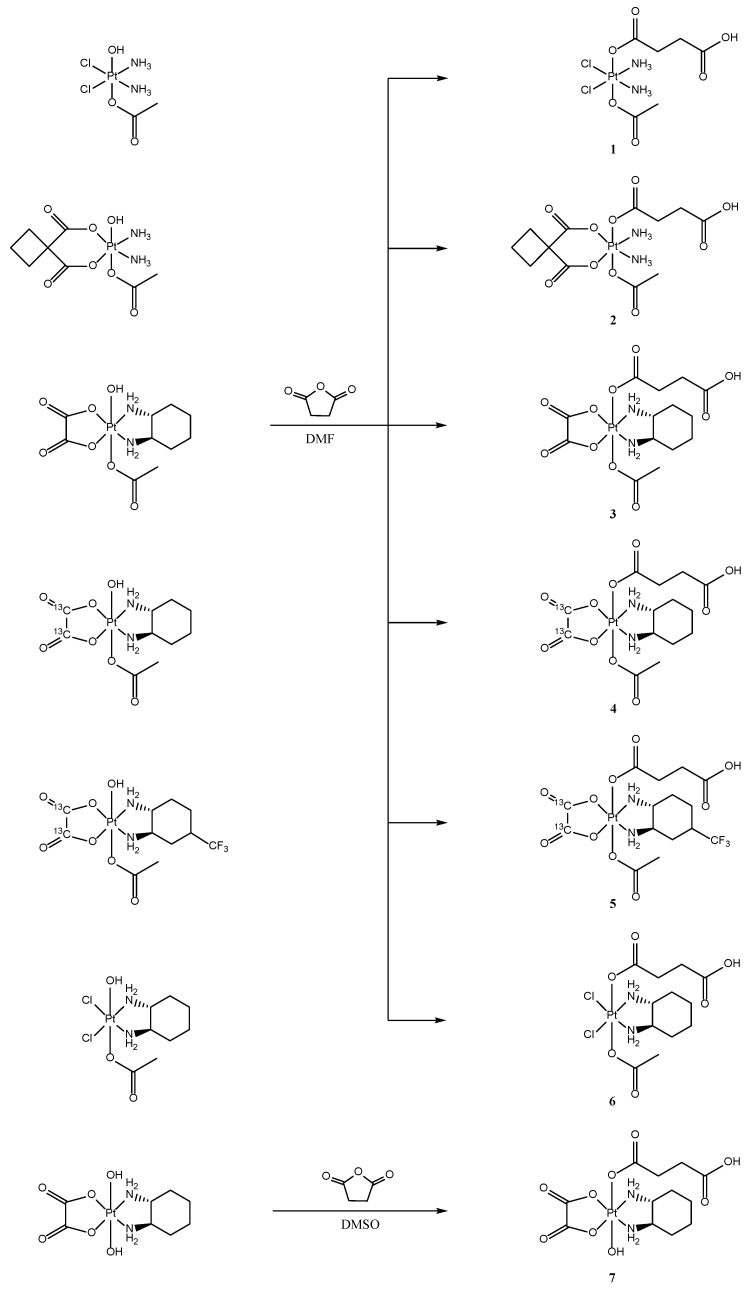
Synthetic pathway leading to platinum(IV) complexes **1**–**7**.

Acetatohydroxidoplatinum(IV) precursor complexes were synthesized according to standard procedures via unsymmetric oxidation with hydrogen peroxide in acetic acid [27]. The trifluoromethyl oxaliplatin analog was synthesized as reported recently [26]. Further reaction with succinic anhydride in absolute DMF resulted in complexes **1**–**6** after purification via RP-HPLC [23]. The synthesis of compound **7** followed a method previously published (Scheme 1) [28].

**Scheme 2:**
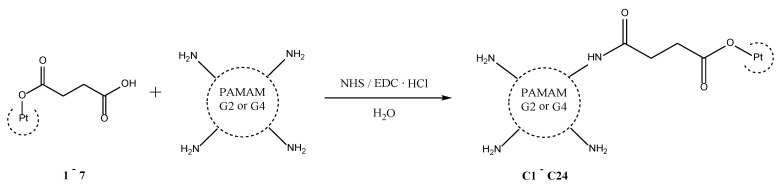
Schematic illustration of the conjugation reaction of platinum(IV) complexes and PAMAM dendrimers via amide bond formation.

The conjugation of platinum(IV) complexes to G2 and G4 PAMAM dendrimers was performed in two steps adapted from a previously published procedure [23]. At first, COOH groups of compounds **1**–**7** were activated with the coupling reagents N-(3-dimethylaminopropyl)-N′-ethylcarbodiimide hydrochloride (EDC·HCl) and N-hydroxysuccinimide (NHS) forming an NHS-ester [29]. The addition of G2 and G4 PAMAM dendrimers containing primary amines as terminal groups resulted in amide bond formations leading to conjugates **C1**–**C24** (Scheme 2). Purification was performed via dialysis against distilled water using dialysis tubings with a molecular weight cut-off (MWCO) of 1 kDa for conjugates of G2 and MWCO of 3.5 kDa for G4, respectively. The final conjugates, **C1**–**C24**, were obtained via lyophilization. Additionally, conjugates with platinum(IV) complex **6** were synthesized. However, analysis by NMR spectroscopy revealed an additional signal in ^195^Pt NMR spectra, probably caused by hydrolysis during the dialysis process. Consequently, these conjugates could not be included in this study. Furthermore, numerous couplings of the G4 PAMAM dendrimer with platinum(IV) complexes **1** and **3** were conducted with different reaction times in order to vary the loading with platinum(IV) complexes (Table 1).

### 3.2. Analysis

Characterization of platinum(IV) complexes **1**–**7** was performed by using multinuclear one- and two-dimensional NMR spectroscopy (^1^H, ^13^C, ^15^N, ^19^F, ^195^Pt) (Supporting Information, Figures S1–S10) and their purity was validated by elemental analysis (>95%). The platinum(IV)-PAMAM conjugates **C1**–**C24** were analyzed by ^1^H and ^195^Pt NMR spectroscopy (Supporting Information, Figures S11–S20). ^195^Pt resonances between 2611 and 3507 ppm are indicative of the presence of platinum(IV) units. As an example, the ^1^H NMR spectra of G4 PAMAM conjugate **C14** and platinum(IV) complex **3** are shown for comparison (Figure S21). ^1^H signals of conjugates in the region above 2.2 ppm result in part from signals of the bound platinum(IV) moiety as well as from signals of the inner and outer part of the dendrimer with peripheral amine groups which are free or bound to the platinum complex. Therefore, complete signal assignment in proton NMR spectra was unfortunately not possible.

Furthermore, ICP-MS was used for the determination of the platinum amount of conjugates **C1**–**C24**. The different platinum(IV) units per dendrimer and the corresponding loading rates are shown in Table 1. The molecular weight of conjugates **C1**–**C24** was calculated based on the molecular weight of the PAMAM dendrimer (according to Sigma-Aldrich: PAMAM G2 = 3256 g/mol, PAMAM G4 = 14214 g/mol) and the addition of the molecular weight of attached platinum(IV) units, while also considering the release of water molecules during the conjugation process (Table 1).

Additionally, pseudo-2D diffusion-ordered spectroscopy (DOSY) spectra were measured for selected compounds, confirming the conjugation of platinum(IV) complexes to PAMAM dendrimers. As an example, an overlay of DOSY spectra of the conjugate **C14**, the unloaded G4 PAMAM dendrimer and platinum(IV) complex **3** is shown in Figure S22 (Supporting Information). The derived diffusion coefficient was used to estimate the average diameter of unloaded G2 and G4 PAMAM dendrimers as well as of conjugates **C1**–**C3** and **C12**–**C14** using the Stokes–Einstein equation and the assumption that the molecules are spherical (Table 2). As expected, the conjugation of platinum(IV) complexes to dendrimers significantly increased the diameter compared to unloaded PAMAM dendrimers. The calculated diameters are consistent with previously published data [23].

Moreover, the reduction behavior of conjugates in comparison to unattached platinum(IV) complexes was investigated by time-dependent ^1^H NMR spectroscopy (Table 3). In general, the reduction behavior is influenced by the nature of the ligands coordinated to the platinum(IV) core. In addition to the axial ligands, the equatorial coordination sphere plays a crucial part as shown by the significantly different rates of reduction of platinum(IV) complexes **1**–**3**, **5**, **6** featuring the same carboxylato ligands in axial position. In accordance with previous studies [30,31], the cisplatin core of substance **1** led to a faster reduction with a reduction half-time of 6 h, whereas only 6% of the carboplatin(IV) analog **2** was reduced after 95 h. Similar to complex **1**, the two chlorido ligands of compound **6** allow fast electron transfer, thereby supporting rapid reduction [32]. The additional trifluoromethyl group in position 4 of the DACH ligand of complex **5** seems to cause a faster reduction in comparison to substance **3**, comparable with the discovered relationship of electron-withdrawing power and rate of reduction for axial ligands [33,34]. Furthermore, the strong effect of axial ligands on the reduction behavior is displayed by the comparison of the two oxaliplatin(IV) analogs **3** and **7**. The amine and carboxylato ligands of complex **3** cannot form bridges with the reducing agent and thus do not support the electron transfer from ascorbate to the platinum(IV) atom in the center. Consequently, only 14% of compound **3** was reduced after 165 h. In contrast, the hydroxido ligand of complex **7** facilitates electron transfer and results in a significantly lower reduction half-time of 29 h, consistent with previously published results [35,36]. The same order of rates of reduction was observed for the conjugates of the respective platinum(IV) complexes, except for **C23**. Generally, all conjugates underwent faster reduction compared to their corresponding platinum(IV) complexes, possibly caused by the increased bulkiness of the axial ligand. A potential connection between bulkiness and facilitated reduction has been reported in the literature [37]. As further expected, the influence between G2 and G4 dendrimers on the reduction behavior is marginal due to the huge distance to the platinum(IV) core. Therefore, no significant difference in the rate of reduction between conjugates of G2 and G4 dendrimers was observed based on the measurements of **C2** and **C13**, respectively.

**Figure 1:**
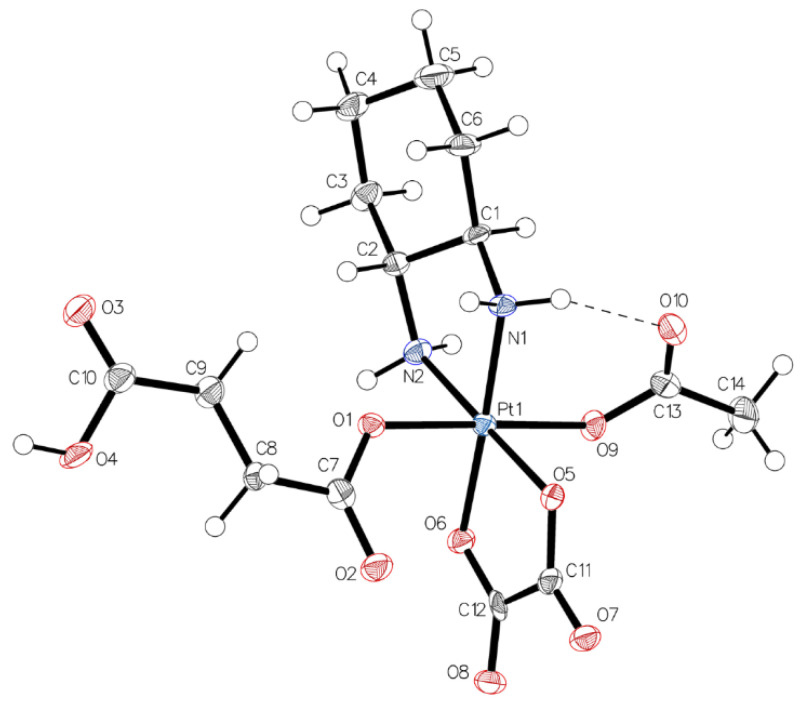
ORTEP view of complex **3**. Asymmetric unit of platinum(IV) complex **3** drawn with 50% displacement ellipsoid with a bond precision for C-C single bonds of 0.0099 Å. The chiral interpretation is done with the help of Flack and Hooft parameters (−0.023, −0.019), and can be determined for C1 (R) and C2 (R). Solvent and disorder (main residue disorder = 4%) were omitted for clarity.

Finally, a single crystal of oxaliplatin analog **3** was obtained from a methanol solution by slow evaporation at room temperature and was analyzed by X-ray diffraction. Complex **3** crystallized in the orthorhombic space group P2_1_2_1_2_1_ and confirmed the octahedral coordination sphere with the platinum(IV) atom in the center (Figure 1). The bidentate oxalato and DACH ligand are in equatorial position with bite angles of 84.74(16)° (O5–Pt1–O6) and 83.59(19)° (N1–Pt1–N2), respectively. The structure is completed by two axially coordinated carboxylato ligands featuring an O1–Pt1–O9 angle of 177.1(2)°. The equatorial Pt–N (Pt1–N1, 2.031(5); Pt1–N2, 2.034(5) Å) and Pt–O (Pt1–O5, 2.010(4); Pt1–O6, 2.009(4) Å) distances are comparable to other oxaliplatin analogs reported in the literature [38], whereas Pt–O bond lengths of carboxylato ligands in axial position were found at 2.004(4) (Pt1–O1) and 2.002(4) Å (Pt1–O9), and are comparable with those of previously published platinum(IV) complexes [39]. Additionally, one intra-molecular hydrogen bond N1–H∙∙∙∙∙O10 of moderate character with a donor–acceptor contact of 2.715 Å (N1∙∙∙∙∙O10) and an angle of 130.1° (N1–H∙∙∙∙∙O10) was found in the solid state. Further details about the crystal structure can be found in the Supporting Information (Tables S1–S3).

### 3.3. Cytotoxicity

Three human cancer cell lines differing in their chemosensitivity were employed for testing the cytotoxic potencies of all compounds: the broadly sensitive ovarian teratocarcinoma cell line CH1/PA-1, the multidrug-resistant non-small cell lung cancer cell line A549 and the colon cancer cell line SW480 with mostly intermediate sensitivity. IC_50_ values interpolated from concentration–effect curves (Supporting Information, Figures S23–S29) are listed in Table 4. This pattern of sensitivity also reflects throughout the data compiled here.

As expected due to their higher inertness, platinum(IV) complexes **1**–**3** are by one to two orders of magnitude less potent than the corresponding platinum(II) drugs cisplatin, carboplatin and oxaliplatin. Of the structural modifications imposed on **3**, only the exchange of oxalate for two chlorido ligands had conspicuous consequences for biological activity: IC_50_ values of the dichlorido analog **6** are 9–26 times lower than those of **3**, depending on the cell line, whereas addition of a CF_3_ substituent to the DACH ligand (in **5**) or replacement of the axial acetato ligand with a hydroxido group (in **7**) yielded minor, if any, changes in cytotoxic potency.

Except for the rather inefficient conjugate **C2** (where, moreover, a comparison with the unconjugated complex **2** is only partially possible, as some IC_50_ values were not even reached), loading of the platinum(IV) complexes onto G2 PAMAM dendrimers (**C1**, **C3**–**C5**) resulted in products with 4–30 times increased cytotoxic potency in absolute numbers. This implies that the products mostly exert at least the effect that could roughly be expected from their degree of platinum(IV) loading (4–9 platinum(IV) units per dendrimer), but even higher effects were observed in some cases (e.g., compare **C4** with **5** in CH1/PA-1 cells).

Apart from a singular exception (conjugate **C23** containing complex **5**), the effects of loaded G4 PAMAM dendrimers are not less remarkable: the majority of **C6**–**C12** (loaded with cisplatin analog **1**) and, even more so, **C14**–**C22** (loaded with oxaliplatin analogs **3** or **4**) is much more to tremendously more potent than could be explained by the mere ratios of platinum(IV) loading (especially compare **C15**, **C20**, **C21** or also **C22** with **3**). Reasons are likely to be multifactorial. It has been reported that dendrimers can enhance membrane permeability and therefore increase the cellular uptake of drugs. The conjugation of anticancer agent paclitaxel to lauryl-modified G3 PAMAM dendrimers led to up to 12-times higher permeability than free paclitaxel in monolayers of the human colon adenocarcinoma cell line Caco-2, as well as in porcine brain endothelial cells [40]. Furthermore, enhanced cellular uptake by a factor of up to 11 was detected with G4 PAMAM dendrimers combined with cisplatin in A2780 ovarian cancer cells compared to free cisplatin [19]. Additionally, a relationship of fast reduction leading to increased cytotoxicity is widely accepted [41]. According to Ref. [19], the accelerated activation of conjugates by reduction based on faster reduction half-times (Table 2) as compared to their corresponding platinum(IV) complexes may play a significant part in increased cytotoxicity. In addition to enhanced permeability, cellular uptake and faster reduction half-times, it is conceivable that synergies between the individual effects of platinum(IV) complex and G4 PAMAM dendrimer additionally contribute to the extraordinary enhancement of cytotoxicity, since even the unloaded G4 PAMAM dendrimer (in contrast to G2) exerts antiproliferative activity in the low micromolar concentration range in all three cell lines. However, detailed investigations are needed to fully understand the mechanism of significantly increased cytotoxicity of platinum(IV)-based PAMAM dendrimer conjugates. Furthermore, it is intriguing that higher loading than the applied minimum of about 10 platinum(IV) units per dendrimer did not necessarily (or, in fact, rather occasionally and under proportionally) lead to further increase in potency, neither in series **C6**–**C12** nor **C14**–**C21**. It is conceivable that reasons are similar to a previously conducted study of half-generation PAMAM dendrimers loaded with cisplatin, in which an incomplete drug release was detected probably caused by intramolecular interactions of platinum complex and dendrimer branches [42].

### 3.4. In Vivo Studies

In order to validate the in vivo efficacy of the platinum(IV)-loaded dendrimer strategy, one representative conjugate of the platinum(IV) series of cisplatin (**C11**), carboplatin (**C13**), and oxaliplatin (**C22**) was tested in the G4 PAMAM dendrimer background. The toxicity tests were performed by tail vein injection (every second day for 3 injections in total) into non-tumor-bearing mice at a dose equimolar to the released platinum(II) species: **C11**, 0.17 mg/20 g, equimolar to 3 mg/kg cisplatin; **C13**, 0.91 mg/20 g, equimolar to 17 mg/kg carboplatin; **C22**, 0.52 mg/20 g; equimolar to 9 mg/kg oxaliplatin. Out of the three tested substances, the cisplatin-based conjugate **C11** showed by far the best tolerability without significant weight loss or profound changes in behavior and showed only temporal mild hair loss. In contrast, oxaliplatin-based conjugate **C22** led in both treated mice to tail necrosis, forcing the termination of this experimental group based on ethical guidelines. The carboplatin-based conjugate **C13** induced moderate weight and strong hair loss. Thus, the cisplatin-based conjugate was further analyzed in more depth. An additional toxicity assay with **C12**, a higher platinum(IV)-loaded conjugate compared to **C11**, was performed at concentrations equimolar to 1.5 and 3 mg/kg cisplatin and the same application scheme as before. Again, no signs of toxicity were observed.

Consequently, **C12** was chosen for the therapy experiment. As the anticancer activity of platinum drugs includes also immunological mechanisms [43], the colon cancer allograft model was used. As a first step, the impact of cisplatin was tested compared to **C12** in CT26 murine colon cancer cells in vitro. Compound **C12** exerted a more than four-fold lower IC_50_ value as compared to cisplatin (**C12**: 0.43 µM; cisplatin: 1.85 µM, Supporting Information, Figure S30), thus resembling data in the human cancer cell models (compare Table 4).

**Figure 2:**
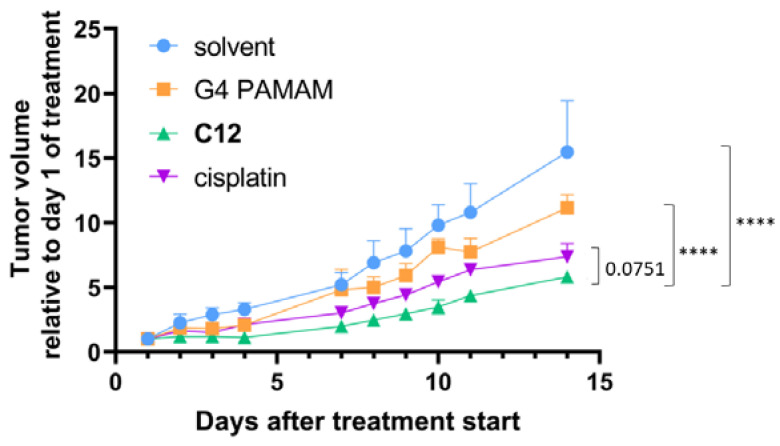
Comparison of the tumor volume development with treatment of solvent, G4 PAMAM, conjugate **C12** and cisplatin in BALB/c mice over 14 days. Significances were determined via Turkey’s multiple comparisons test with the following abbreviations: **** *p* < 0.0001.

**Figure 3:**
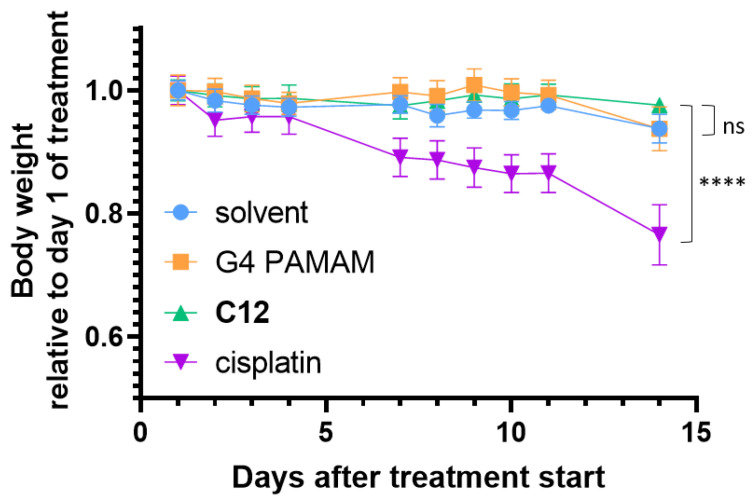
Comparison of the body weight development with treatment of solvent, G4 PAMAM, conjugate **C12** and cisplatin in BALB/c mice over 14 days. Significances were determined via Turkey’s multiple comparisons test with the following abbreviations: ns = not significant, **** *p* < 0.0001.

Based on this higher cytotoxic activity and clearly better tolerability in non-tumor-bearing animals, the efficacy of **C12** (7.5 mg/kg: equimolar to 3 mg/kg cisplatin) was compared with the respective dose of free cisplatin (3 mg/kg) and unloaded G4 PAMAM dendrimer in CT26-allograft-bearing mice. Drugs were given intravenously for two weeks twice a week. The impact of the different treatment groups as compared to solvent control on tumor volume and body weight until day 14 (loss of the first mouse in the solvent group due to big tumor size) are given in Figure 2 and Figure 3, respectively. All treatments significantly reduced tumor volume as compared to the solvent control with a maximum tumor growth inhibition effect of 37% for unloaded G4 PAMAM dendrimer, 47.6% for cisplatin, as well as 65.6% for **C12**. In addition, concerning the tumor growth curves, the strongest activity was exerted by **C12**, although the difference between free cisplatin and **C12** did not reach statistical significance in the multiple comparison tests (*p* = 0.075). Concerning toxicity, neither free G4 PAMAM dendrimer nor **C12** led to any loss of body weight. In contrast, free cisplatin at the maximal tolerated dose of 3 mg/kg significantly reduced body weight as compared to all other experimental groups with a maximal loss of body weight of around 20% at day 14 of treatment. This data strongly suggests an improved therapeutic window for cisplatin when given as a PAMAM-dendrimer-based nano-formulation.

**Figure 4:**
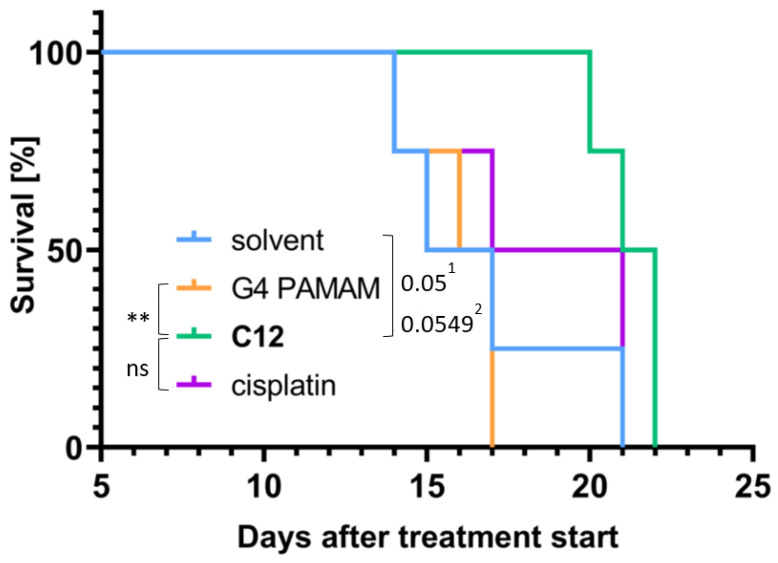
Comparison of the survival time with treatment of solvent, G4 PAMAM, conjugate **C12** and cisplatin in BALB/c mice over 14 days. Significances were determined via log-rank test (LRT) and Gehan–Breslow-Wilcoxon test (GBWT) with the following abbreviations: ns = not significant, ** *p* < 0.01. ^1^ with GBWT. ^2^ with LRT.

The promising effects further translate into a prolongation trend of animal survival (Figure 4). While in the cisplatin group, weight loss was critical in addition to tumor necrosis, even smaller tumors tended to get necrotic and broke up in **C12**-treated animals, making the sacrifice of the animals necessary due to ethical guidelines. In contrast to the cisplatin and G4 PAMAM dendrimer treatment arms, only the anticancer activity of **C12** showed a trend towards longer survival (*p*-value of 0.0549 in log-rank and 0.05 in Gehan–Breslow–Wilcoxon test) in the direct comparison with the solvent control arm. A more extended analysis of different doses and schedules is needed to optimize the therapeutic effects of **C12,** but limit the massive necrotizing effects leading to the termination of the experiment due to tumor ulceration.

**Figure 5:**
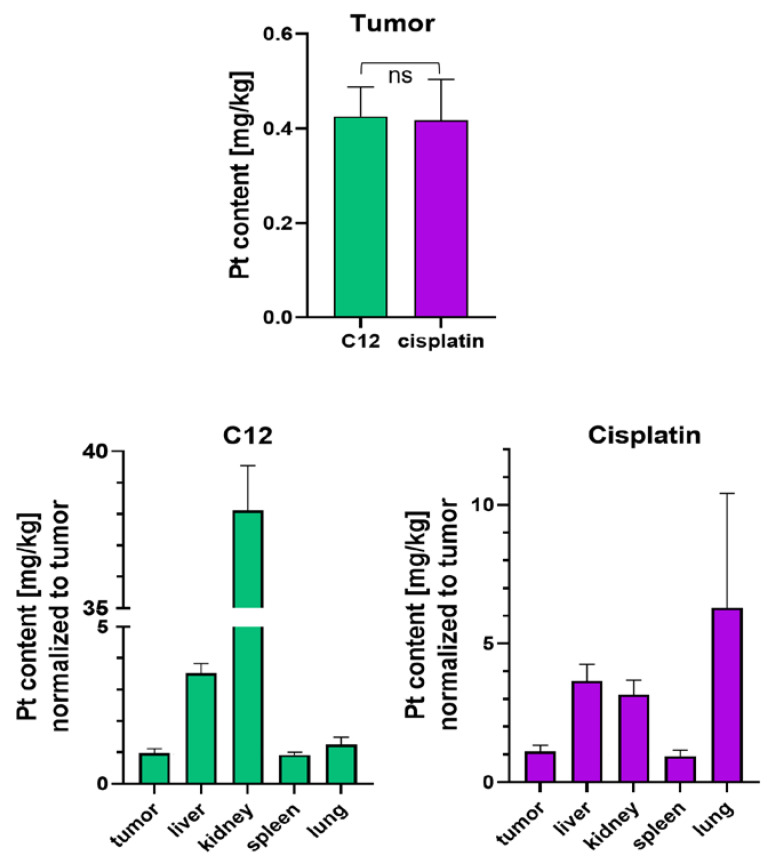
Comparison of the platinum accumulation in tumors and different organs of mice treated with conjugate **C12** (green) and cisplatin (violet) 24 h after second application measured by ICP-MS. Significances were determined via Turkey’s multiple comparisons test with the following abbreviation: ns = not significant.

In addition to tumor growth experiments, the tumor and organ distribution of platinum in mice treated with cisplatin or **C12** were determined by ICP-MS (Figure 5). Unexpectedly, the platinum levels in the tumor did not differ significantly 24 h after the second dosing. In contrast, **C12** led to high platinum levels in the serum at all three time points, while blood cells contained enhanced platinum contents in the case of cisplatin treatment (Supporting Information, Figure S31). This suggests a lower clearance and reduced local interaction with blood cells of **C12**, as compared to cisplatin. In organ distribution (Figure 5), **C12** treatment led to a massive platinum accumulation in the kidney as compared to all other organs, while in the case of cisplatin, higher levels were detected in lung tissue, however, with great inter-individual differences. These data strongly suggest that the PAMAM dendrimer formulation leads to trapping of the nano-formation probably based on filtration in the glomerulus and reabsorbed in the lumen of the proximal tubule. Renal excretion and glomerular filtration are typical for ≤G4 PAMAM dendrimers and in general for nanoparticles smaller than 5 nm [44,45]. The conjugation of complex **1** to G4 PAMAM dendrimers considerably increased the polymer diameter of **C14** from about 4.5 to 5.4 nm. Additionally, the influence of the molecular weight of nanoparticles on the biodistribution behavior was reported previously. Nanoparticles <20 kDa primarily undergo renal clearance, whereas bigger molecules show longer blood circulation times and a shift towards clearance by the reticuloendothelial system [46]. Based on a particle size of over 5 nm and a molecular weight of 36.5 kDa of conjugate **C12**, the renal accumulation is rather unexpected and needs to be investigated in more detail. However, it is obvious that, despite these unwanted conditions, **C12** exerted a comparable tumor accumulation to cisplatin, a tendency to enhance anticancer activity, and an improved therapeutic window. This strongly suggests further modifications of the novel cisplatin dendrimeric remedy to ameliorate kidney accumulation and, in parallel, enhance tumor response. On the contrary, the efficient kidney accumulation of this dendrimer preparation might also be considered in kidney-specific drug delivery approaches [47,48,49].

## 4. Conclusions

In the present study, an alternative antitumor strategy was presented by conjugating various platinum(IV)-analogs of cisplatin, carboplatin and oxaliplatin to the surface of G2 and G4 PAMAM dendrimers. Twenty-four novel conjugates were synthesized and characterized by ^1^H and ^195^Pt NMR spectroscopy, as well as DOSY measurements substantiating the successful conjugation. Reduction behavior analysis revealed a significantly faster reduction of all conjugates in comparison to their corresponding platinum(IV) complexes, probably caused by the increased bulkiness of the axial ligands of the conjugates. The accelerated reduction of the conjugates may also, amongst others, be responsible for improved cytotoxicity of the conjugates. Specifically, the conjugation of platinum(IV) complexes to G4 PAMAM dendrimers resulted in IC_50_ values in the low micro- to the nanomolar range, tremendously lower compared to the corresponding platinum(IV) or even platinum(II) complexes. Remarkably, an oxaliplatin(IV)-based conjugate even reached an IC_50_ value of 780 ± 260 pM in the CH1/PA-1 cancer cell line.

Furthermore, the cisplatin(IV)-based conjugate **C12** was investigated in vivo in CT26-allograft-bearing mice due to its best toxicological profile. Concentrations of the conjugate equimolar to 3 mg/kg cisplatin (maximum tolerated dosage [50]) were very well tolerated and even higher doses could be considered in further investigations. Additionally, biodistribution was analyzed in tumor-bearing mice 24 h after the second application. Unexpectedly, increased accumulation in the kidney was detected despite a higher cut-off molecular weight and particle size of **C12.** It needs to be investigated in more detail, whether **C12** behaves like nanoparticles with a hydrodynamic diameter between 5 nm and 100 nm efficiently crossing the endothelial layer, but blocked by the glomerular basement membrane [51]. Due to the connection of preferred renal excretion with small molecular weights and particle sizes as well as cationic surface charges, supporting attraction to the negatively charged endothelial and podocyte glycocalyx, the use of amine-terminated PAMAM dendrimers >G4 could be considered to reduce renal accumulation. Nevertheless, besides increased molecular weight and size, higher generations of cationic PAMAM dendrimers (>G4) are accompanied by a sharp increase in cytotoxicity based on their increased positive charge density [52,53]. However, additional surface modifications (e.g., PEGylation) of the terminal positively charged amines under physiological conditions could reduce undesired toxicities and further decrease the preference for renal excretion [54,55].

The following anticancer activity experiments revealed a maximum tumor growth inhibition effect of 65.6% for **C12** compared to 47.6% for cisplatin. Additionally, the treatment with **C12** had no negative influence on body weight, whereas cisplatin application led to a maximal loss of body weight of around 20%, enabling an improved therapeutic window for **C12** compared to cisplatin. Furthermore, a trend of extended animal survival with the treatment of **C12** could be observed compared to the solvent control and cisplatin group.

Finally, it can be concluded that the combination of platinum(IV) complexes coupled with PAMAM dendrimers enables a promising approach to further improve existing anticancer therapy. The full potential could be exploited by further investigations of the therapeutic window as well as adjustments on the surface of PAMAM dendrimers to further optimize pharmacological properties.

## Figures and Tables

**Table 1:** Overview of the composition of conjugates **C1**–**C24** including platinum(IV) units per PAMAM dendrimer, average loading rates and molecular weights (MW).

Sample	Pt(IV)	PAMAM	Number of Terminal NH_2_ Moieties	Pt(IV) Units per PAMAM	Average Pt(IV) Loading [%]	MW [kDa]
**C1**	**1**	G2	16	5.97	37.31	6.0
**C2**	**2**	G2	16	4.82	30.13	5.8
**C3**	**4**	G2	16	5.93	37.06	6.6
**C4**	**5**	G2	16	4.38	27.38	6.0
**C5**	**7**	G2	16	7.66	47.88	7.2
**C6**	**1**	G4	64	8.70	13.59	18.2
**C7**	**1**	G4	64	9.87	15.42	18.7
**C8**	**1**	G4	64	10.20	15.94	18.9
**C9**	**1**	G4	64	11.03	17.23	19.3
**C10**	**1**	G4	64	13.57	21.20	20.4
**C11**	**1**	G4	64	38.61	60.33	31.9
**C12**	**1**	G4	64	48.63	75.98	36.5
**C13**	**2**	G4	64	30.27	47.30	30.2
**C14**	**3**	G4	64	8.41	13.14	18.9
**C15**	**3**	G4	64	10.76	16.81	20.2
**C16**	**3**	G4	64	12.18	19.03	21.0
**C17**	**3**	G4	64	13.14	20.53	21.5
**C18**	**3**	G4	64	17.27	26.98	23.8
**C19**	**3**	G4	64	21.62	33.78	26.2
**C20**	**3**	G4	64	27.39	42.80	29.4
**C21**	**3**	G4	64	55.51	86.73	45.0
**C22**	**4**	G4	64	24.29	37.95	27.8
**C23**	**5**	G4	64	15.23	23.80	23.7
**C24**	**7**	G4	64	18.60	29.06	23.8

**Table 2:** Estimated diameters using DOSY spectra and Stokes–Einstein equation of G2 and G4 PAMAM dendrimers as well as of conjugates **C1**–**C3** and **C12**–**C14**.

Sample	Pt(IV)	PAMAM	Diameter [Å]	Diameter Increase [%]
G2 PAMAM	-	G2	~27.9	-
G4 PAMAM	-	G4	~44.4	-
**C1**	**1**	G2	~30.4	~9
**C2**	**2**	G2	~36.9	~32
**C3**	**3**	G2	~36.5	~31
**C12**	**1**	G4	~54.4	~23
**C13**	**2**	G4	~63.6	~43
**C14**	**3**	G4	~60.0	~35

**Table 3:** Overview of the reduction half-times of platinum(IV) complexes and representative conjugates at ambient temperature (ratio complex: ascorbic acid = 1:25). Due to long reduction half-times, some measurements were stopped before reaching the reduction half-time, and the percentage of the reduced species at this point is mentioned in brackets.

Sample	Pt(IV)	PAMAM	Reduction Half-Time [h]
**1**	**1**	-	~6
**2**	**2**	-	>95 (~6%)
**3**	**3**	-	>165 (~14%)
**5**	**5**	-	>93 (~23%)
**6**	**6**	-	~27
**7**	**7**	-	~29
**C2**	**2**	G2	>92 (~37%)
**C11**	**1**	G4	~3
**C13**	**2**	G4	>95 (~42%)
**C22**	**4**	G4	~45
**C23**	**5**	G4	~65
**C24**	**7**	G4	n/a

**Table 4:** Cytotoxicity of platinum(IV) complexes **1**–**3** and **5**–**7** as well as their dendrimer conjugates **C1**–**C24** in comparison with parental platinum(II) drugs in three human cancer cell lines. Mean IC_50_ values are indicated ± standard deviations and are obtained from at least three independent MTT assays (96 h exposure) and pertain to dendrimer concentration for **C1**–**C24**.

Sample	Pt(IV)	PAMAM	Pt(IV) Units per PAMAM	IC_50_ [µM] A549	IC_50_ [µM] CH1/PA-1	IC_50_ [µM] SW480
G2 PAMAM [23]	-	G2	-	>50	67 ± 19	>50
G4 PAMAM [23]	-	G4	-	8.2 ± 2.4	1.5 ± 0.4	6.2 ± 1.1
Cisplatin [24]	-	-	-	3.8 ± 1.0	0.073 ± 0.001	2.3 ± 0.2
Carboplatin [24]	-	-	-	38 ± 3	0.79 ± 0.11	42 ± 10
Oxaliplatin [24]	-	-	-	0.98 ± 0.21	0.18 ± 0.01	0.29 ± 0.05
**1** [28]	**1**	-	-	99 ± 17	1.2 ± 0.5	47 ± 11
**2** [28]	**2**	-	-	>200	16 ± 6	>200
**3** [28]	**3**	-	-	70 ± 29	4.1 ± 0.6	22 ± 8
**5**	**5**	-	-	54 ± 6	6.0 ± 0.9	33 ± 5
**6**	**6**	-	-	2.8 ± 0.1	0.44 ± 0.10	0.85 ± 0.14
**7**	**7**	-	-	66 ± 2	5.2 ± 0.8	20 ± 3
**C1**	**1**	G2	5.97	10.7 ± 0.3	0.31 ± 0.10	6.4 ± 1.3
**C2**	**2**	G2	4.82	>200	11 ± 2	122 ± 8
**C3**	**4**	G2	5.93	6.3 ± 1.3	0.47 ± 0.09	1.7 ± 0.7
**C4**	**5**	G2	4.38	9.9 ± 0.8	0.20 ± 0.06	2.3 ± 0.4
**C5**	**7**	G2	7.66	4.0 ± 0.2	0.22 ± 0.02	0.98 ± 0.18
**C6**	**1**	G4	8.70	1.2 ± 0.3	0.015 ± 0.004	0.42 ± 0.03
**C7**	**1**	G4	9.87	2.5 ± 0.5	0.029 ± 0.004	1.3 ± 0.2
**C8**	**1**	G4	10.20	1.1 ± 0.2	0.0084 ± 0.0036	0.31 ± 0.12
**C9**	**1**	G4	11.03	1.3 ± 0.2	0.011 ± 0.003	0.51 ± 0.05
**C10**	**1**	G4	13.57	1.2 ± 0.2	0.012 ± 0.003	0.41 ± 0.09
**C11**	**1**	G4	38.61	1.4 ± 0.1	0.013 ± 0.001	0.71 ± 0.06
**C12**	**1**	G4	48.63	0.69 ± 0.08	0.0034 ± 0.0016	0.25 ± 0.18
**C13**	**2**	G4	30.27	9.0 ± 1.8	0.025 ± 0.010	2.5 ± 1.8
**C14**	**3**	G4	8.41	0.30 ± 0.05	0.017 ± 0.003	0.072 ± 0.007
**C15**	**3**	G4	10.76	0.092 ± 0.004	0.0078 ± 0.0010	0.038 ± 0.006
**C16**	**3**	G4	12.18	0.20 ± 0.02	0.013 ± 0.001	0.065 ± 0.017
**C17**	**3**	G4	13.14	0.85 ± 0.28	0.039 ± 0.013	0.27 ± 0.08
**C18**	**3**	G4	17.27	1.8 ± 0.3	0.086 ± 0.019	0.50 ± 0.18
**C19**	**3**	G4	21.62	0.24 ± 0.06	0.011 ± 0.003	0.077 ± 0.021
**C20**	**3**	G4	27.39	0.052 ± 0.016	0.0024 ± 0.0006	0.022 ± 0.006
**C21**	**3**	G4	55.51	0.14 ± 0.02	0.0067 ± 0.0005	0.036 ± 0.002
**C22**	**4**	G4	24.29	0.031 ± 0.006	0.00078 ± 0.00026	0.0062 ± 0.0012
**C23**	**5**	G4	15.23	27 ± 1	0.37 ± 0.09	6.7 ± 0.7
**C24**	**7**	G4	18.60	0.191 ± 0.004	0.0069 ± 0.0007	0.056 ± 0.003

## Data Availability

The data presented in this study are available in the supplementary material.

## Supplementary Materials

The following supporting information can be downloaded at https://www.mdpi.com/article/10.3390/pharmaceutics15051515/s1, Figures S1–S10: NMR Spectra of Platinum(IV) Complexes **5**–**7**; Figures S11–S22: NMR Spectra of selected conjugates; Tables S1–S3: X-Ray diffraction analysis; Figures S23–S30: concentration–effect curves; Figure S31: in vivo data. References [56,57,58,59,60,61,62] are cited in the supplementary materials.
