# Supplementary material for: Combination of Drug Delivery Properties of PAMAM Dendrimers and Cytotoxicity of Platinum(IV) Complexes—A More Selective Anticancer Treatment?
Source: Pharmaceutics. 2023 May 17;15(5):1515. doi: 10.3390/pharmaceutics15051515 (PMC10221222; doi:10.3390/pharmaceutics15051515)
Supplement: Supplementary file 1 [file pharmaceutics-15-01515-s001.zip › pharmaceutics-2391671-supplementary.pdf]

Supporting Information

Combination of Drug Delivery Properties of PAMAM Dendrimers and Cytotoxicity of Platinum(IV) Complexes—A More Selective Anticancer Treatment?

Yvonne Lerchbammer-Kreith [1], Michaela Hejl [1], Petra Vician [2], Michael A. Jakupec [1,3], Walter Berger [2,3], Mathea S. Galanski [1,*] and Bernhard K. Keppler [1,3,*]

[1] Institute of Inorganic Chemistry, Faculty of Chemistry, University of Vienna, Waehringer Strasse 42, 1090 Vienna, Austria
[2] Center for Cancer Research and Comprehensive Cancer Center, Medical University of Vienna, Borschkegasse 8a, 1090 Vienna, Austria
[3] Research Cluster "Translational Cancer Therapy Research", University of Vienna, Waehringer Strasse 42, 1090 Vienna, Austria
* Correspondence: mathea.galanski@univie.ac.at (M.S.G.); bernhard.keppler@univie.ac.at (B.K.K.)

Table of Contents

1. **NMR Spectra of Platinum(IV) Complexes 5-7** 3
2. **NMR Spectra of Selected Conjugates** 9
3. **X-Ray Diffraction Analysis** 15
4. **Concentration-Effect Curves** 19
5. **In Vivo Data** 27
6. **References** 28

1. NMR Spectra of Platinum(IV) Complexes 5-7

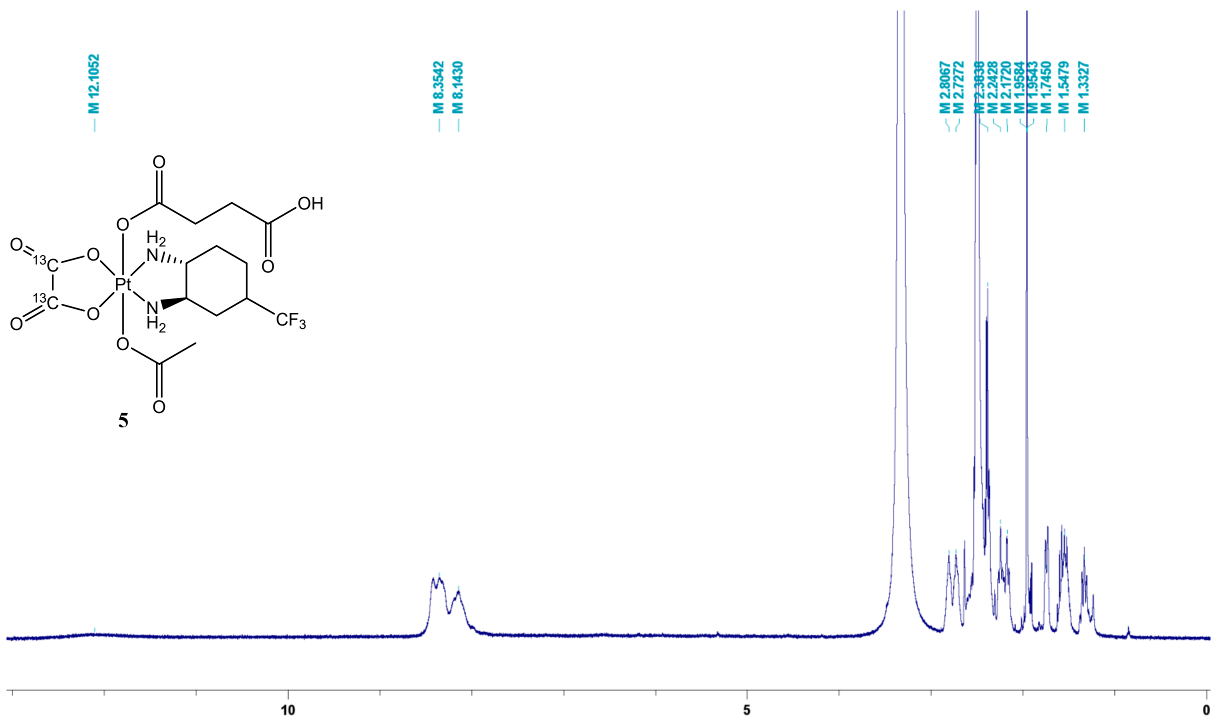

Figure S1. ^{1}H NMR spectrum of complex **5** in d_6-DMSO.

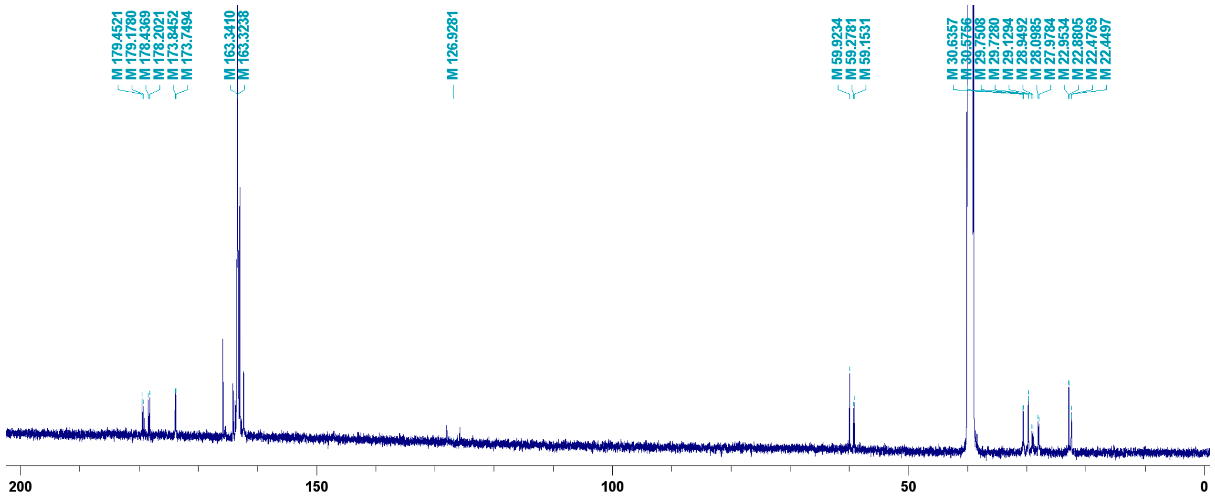

Figure S2. ^{13}C NMR spectrum of complex **5** in d_6-DMSO.

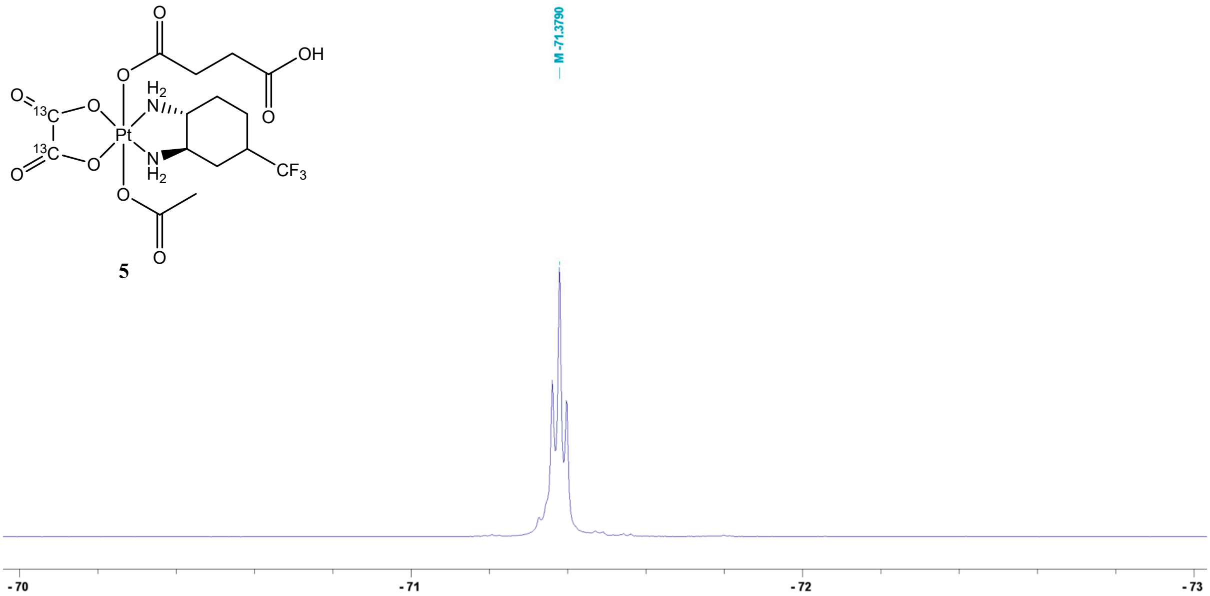

Figure S3. ^{19}F NMR spectrum of complex **5** in d_6-DMSO.

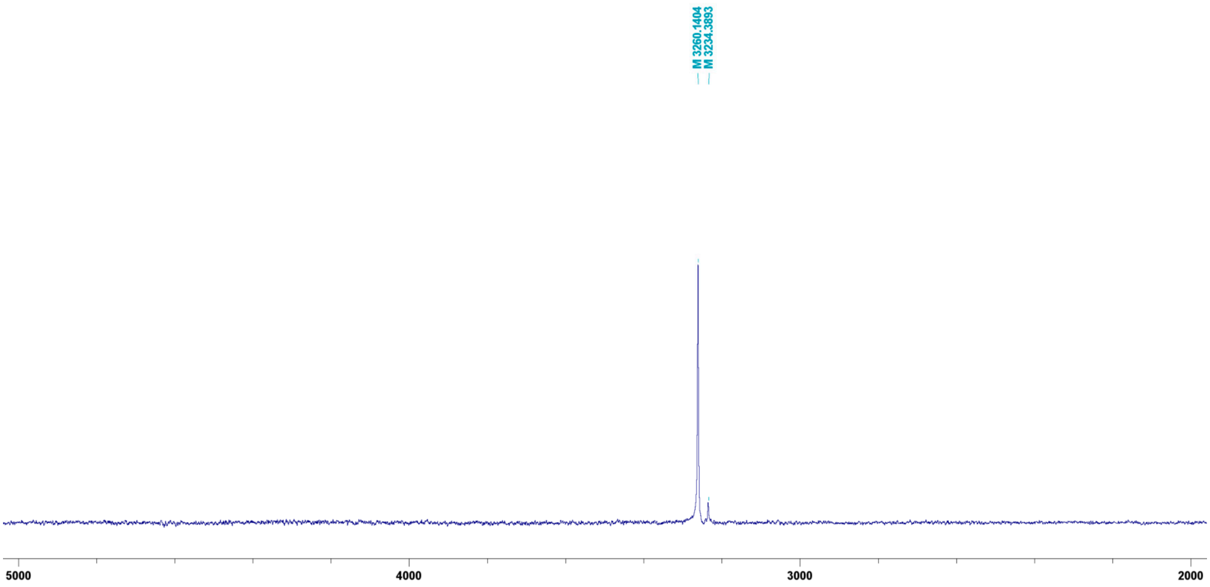

Figure S4. ^{195}Pt NMR spectrum of complex **5** in d_6-DMSO.

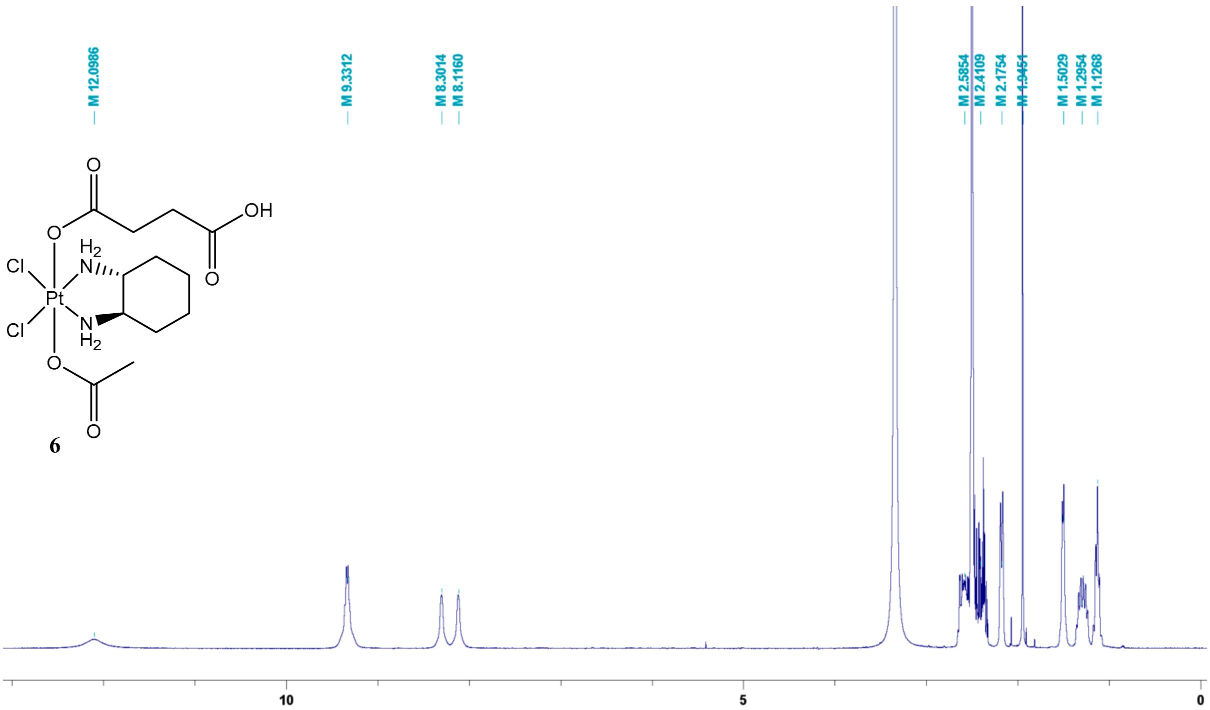

Figure S5. ^{1}H NMR spectrum of complex **6** in d_6-DMSO.

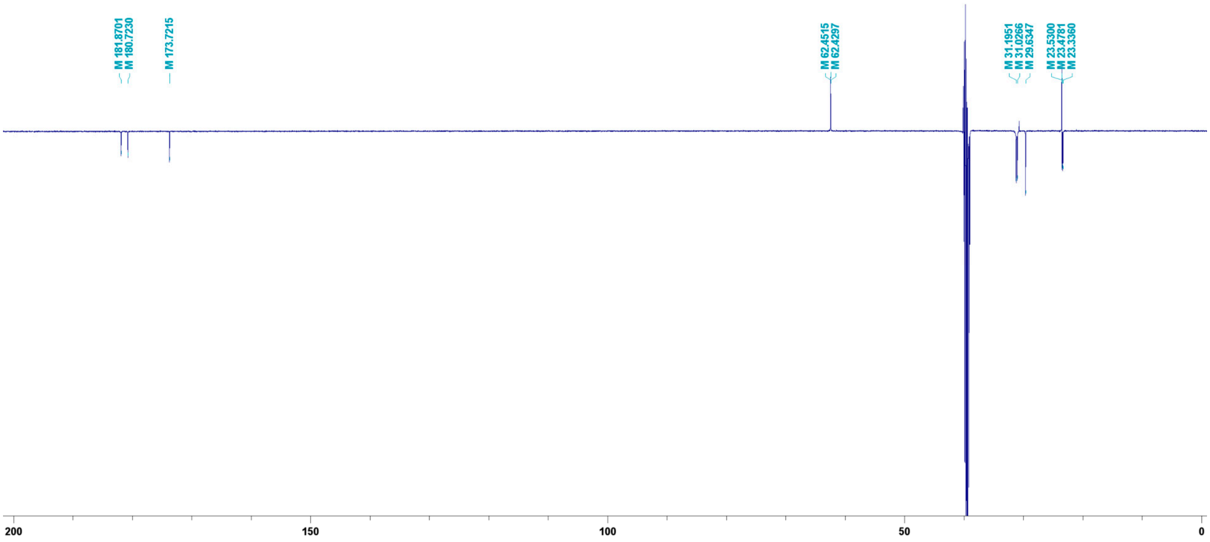

Figure S6. ^{13}C NMR spectrum of complex **6** in d_6-DMSO.

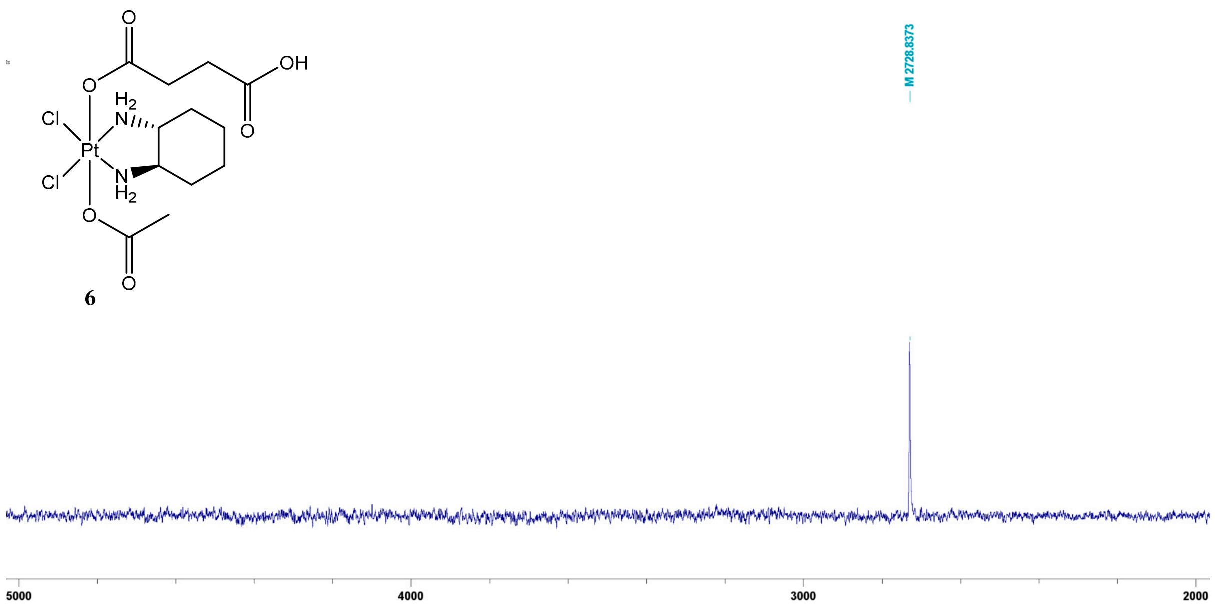

Figure S7. ^{195}Pt NMR spectrum of complex **6** in d_6-DMSO.

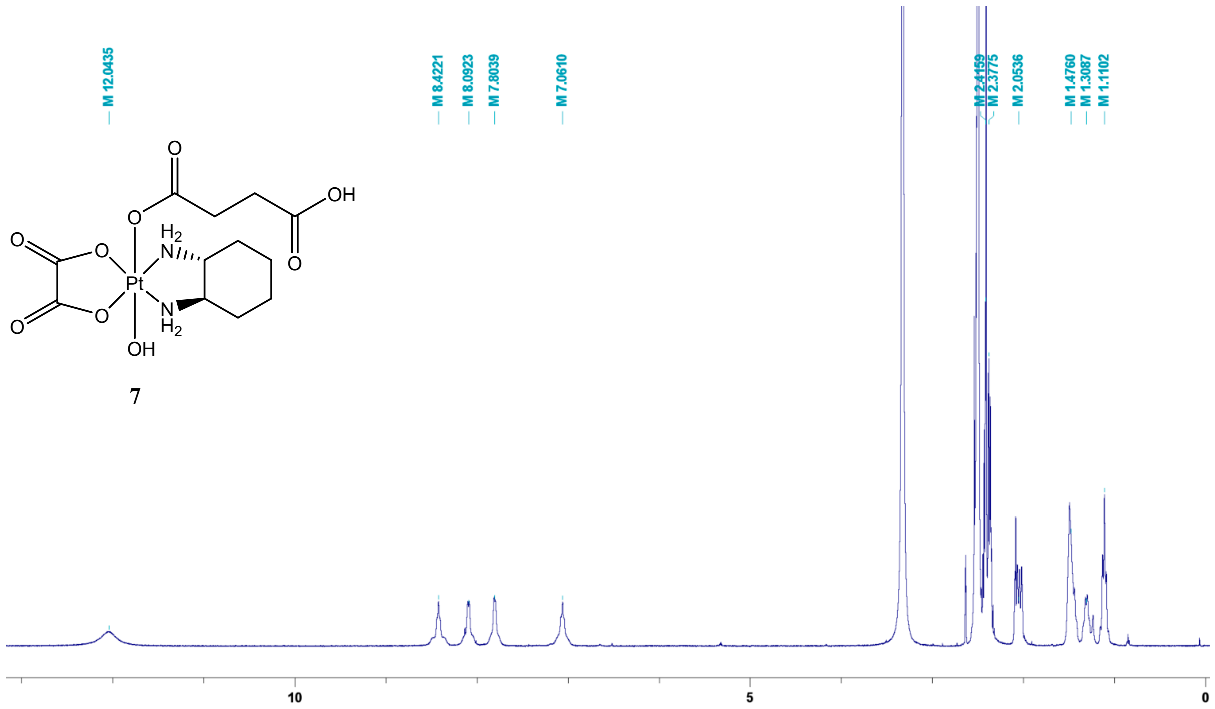

Figure S8. ^{1}H NMR spectrum of complex **7** in d_6-DMSO.

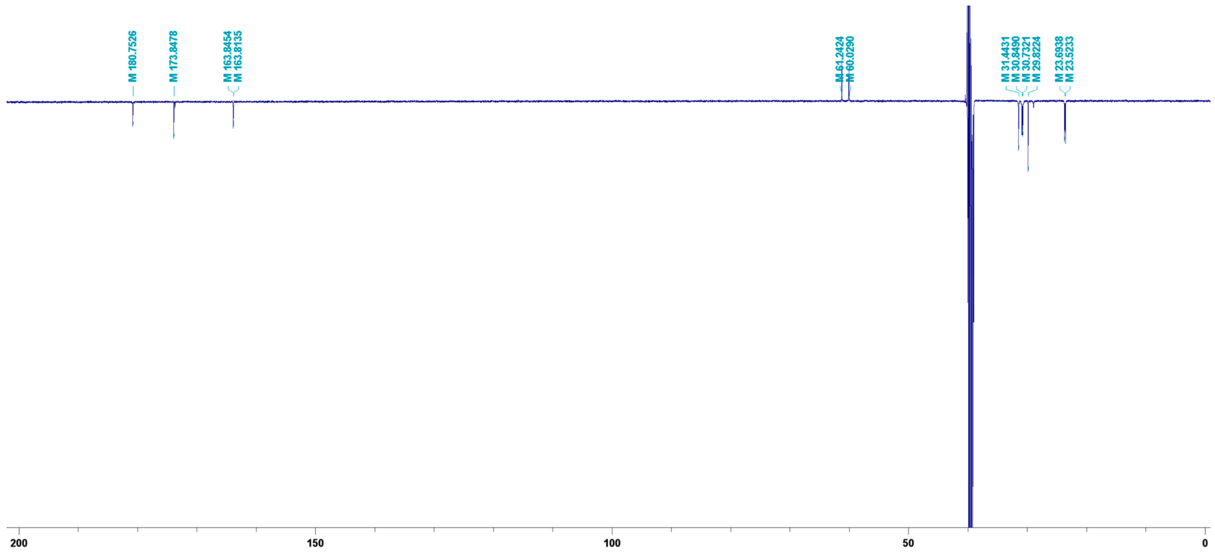

Figure S9. ^{13}C NMR spectrum of complex **7** in d_6-DMSO.

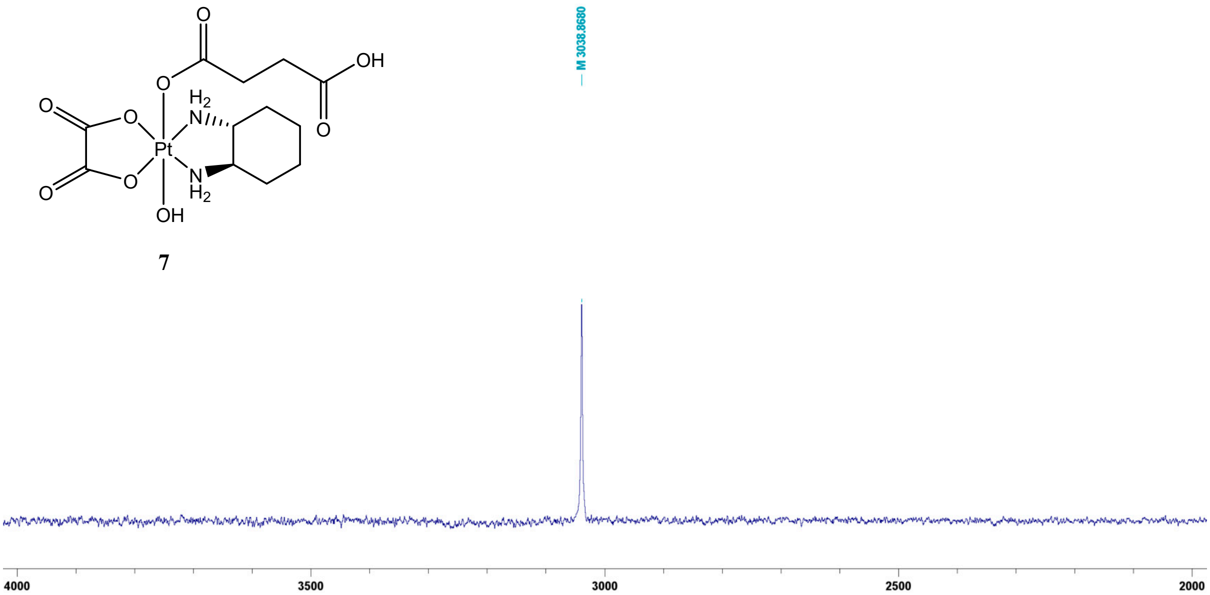

Figure S10. ^{195}Pt NMR spectrum of complex **7** in d_6-DMSO.

2. NMR Spectra of Selected Conjugates

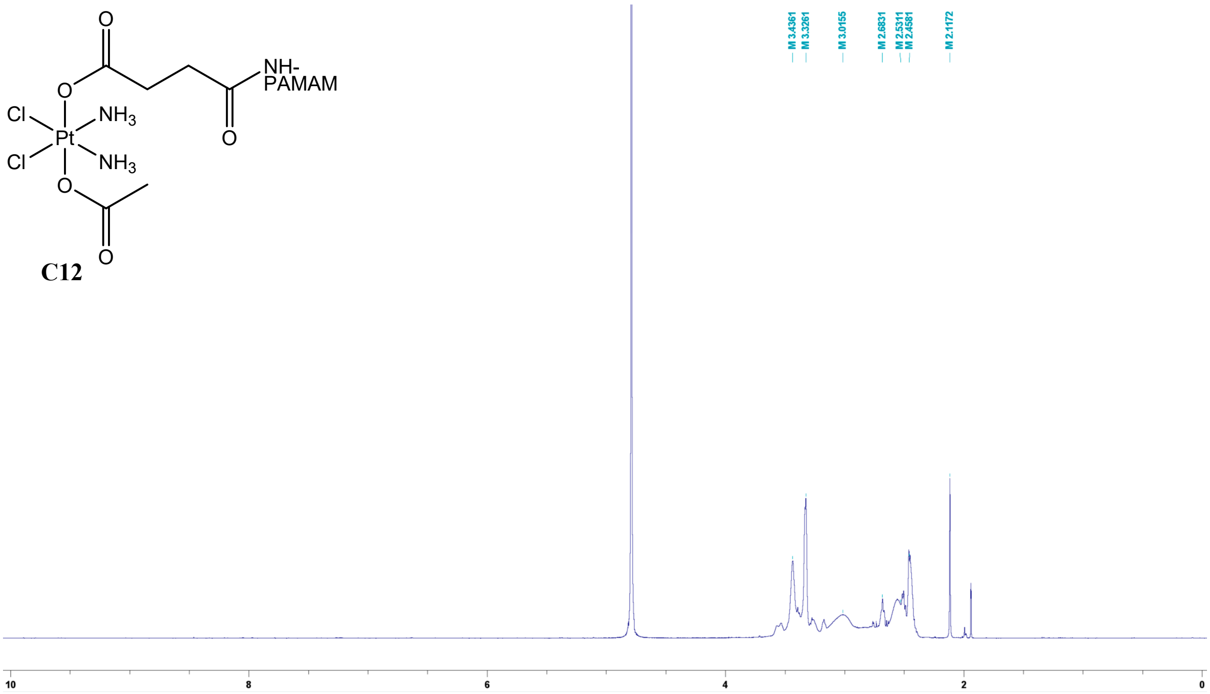

C12

Figure S11. ^{1}H NMR spectrum of conjugate **C12** in D_2O.

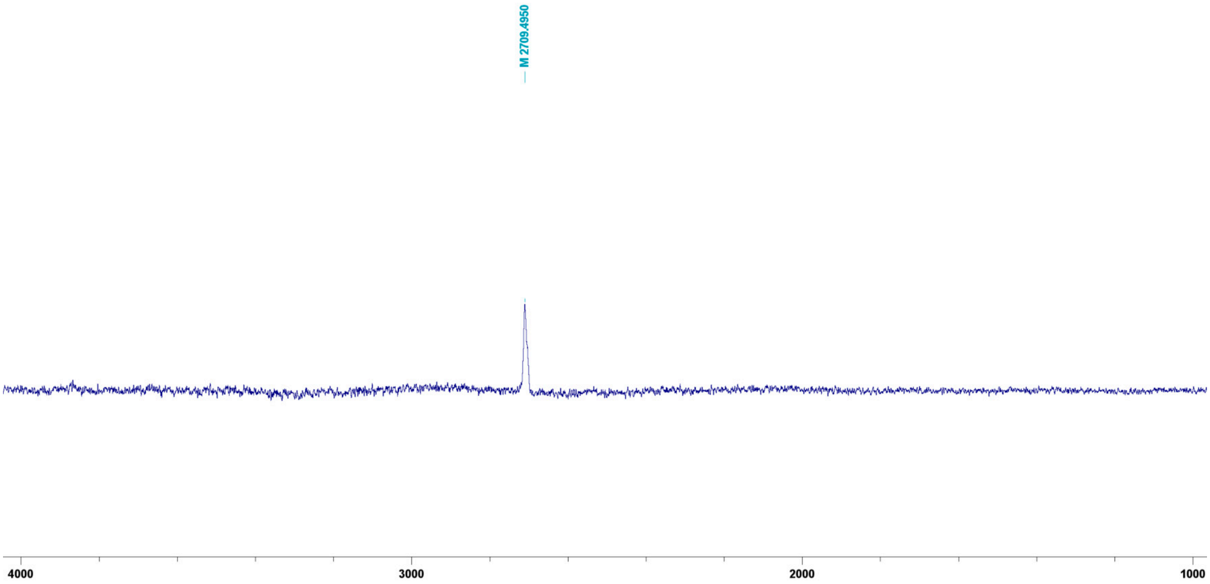

Figure S12. ^{195}Pt NMR spectrum of conjugate **C12** in D_2O.

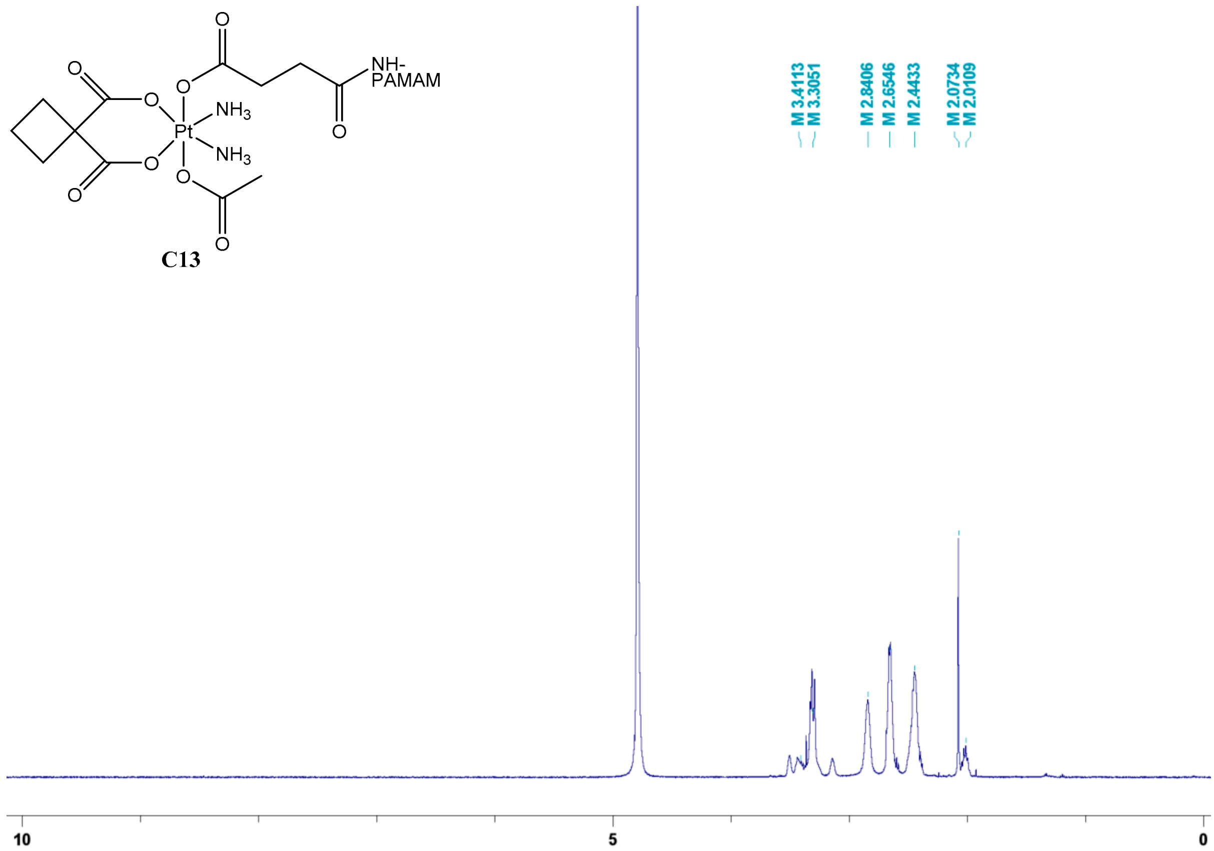

Figure S13. ^{1}H NMR spectrum of conjugate **C13** in D_2O.

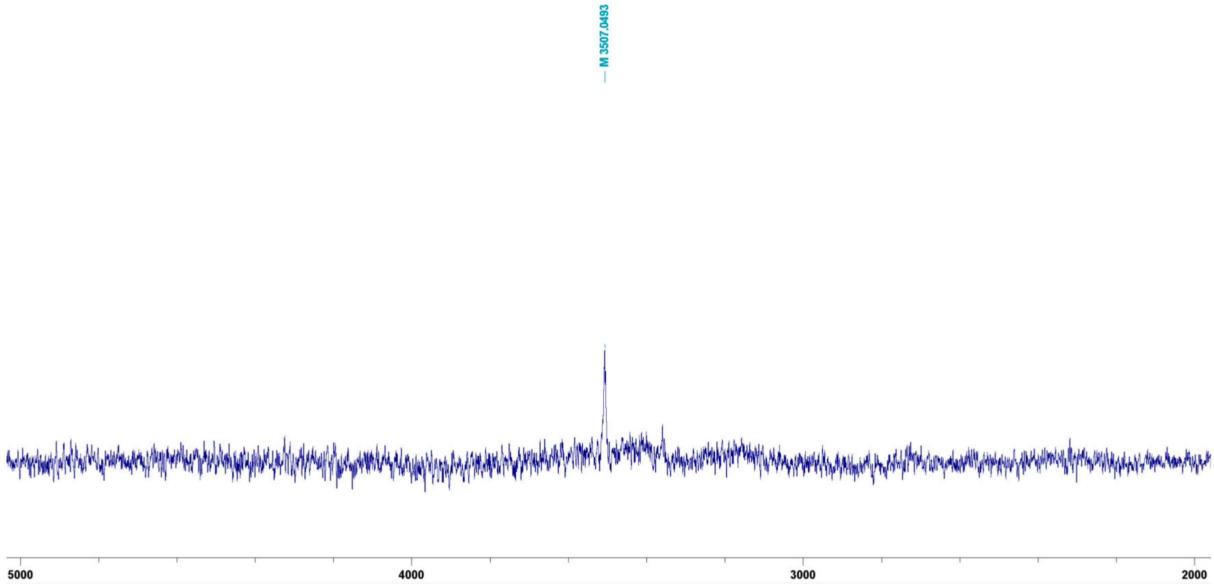

Figure S14. ^{195}Pt NMR spectrum of conjugate **C13** in D_2O.

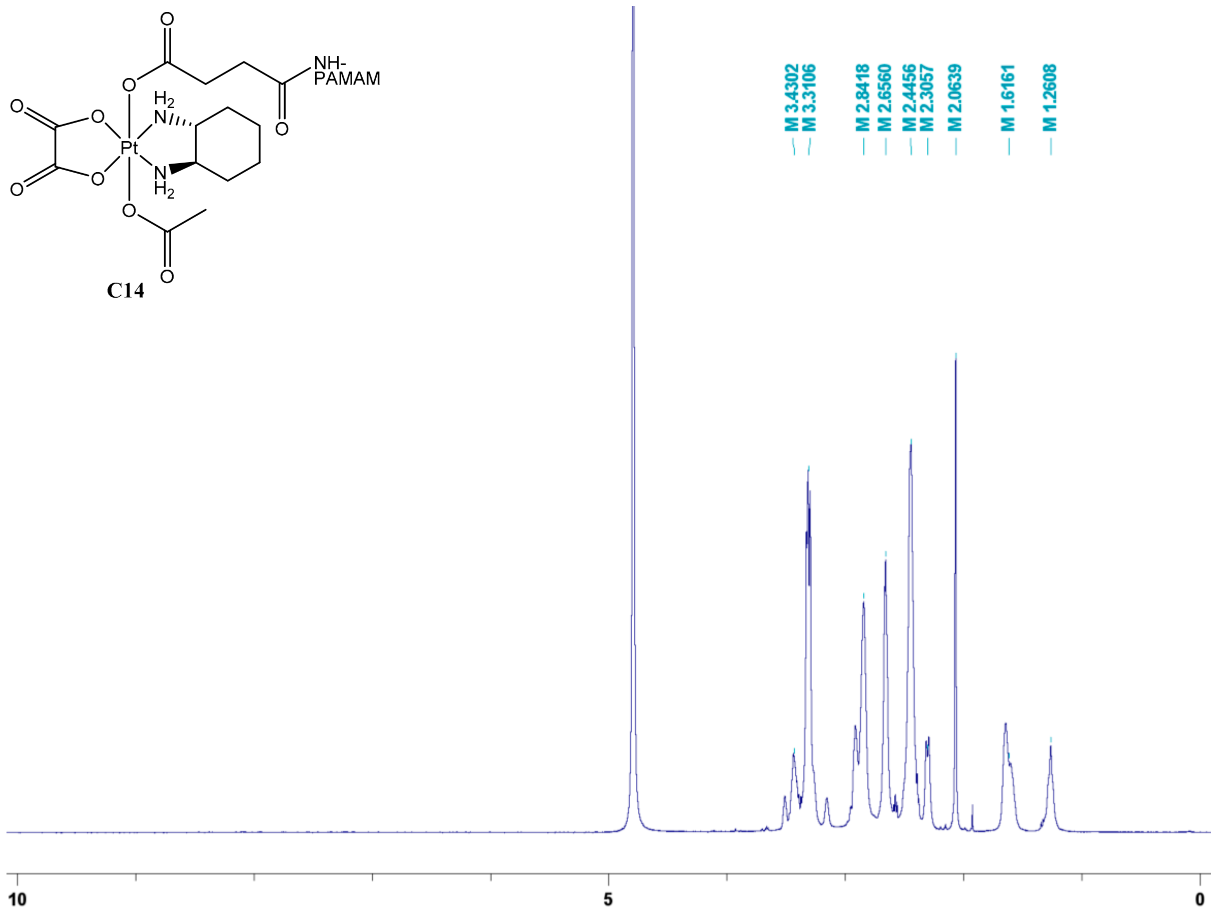

C14

Figure S15. ^{1}H NMR spectrum of conjugate **C14** in D_2O.

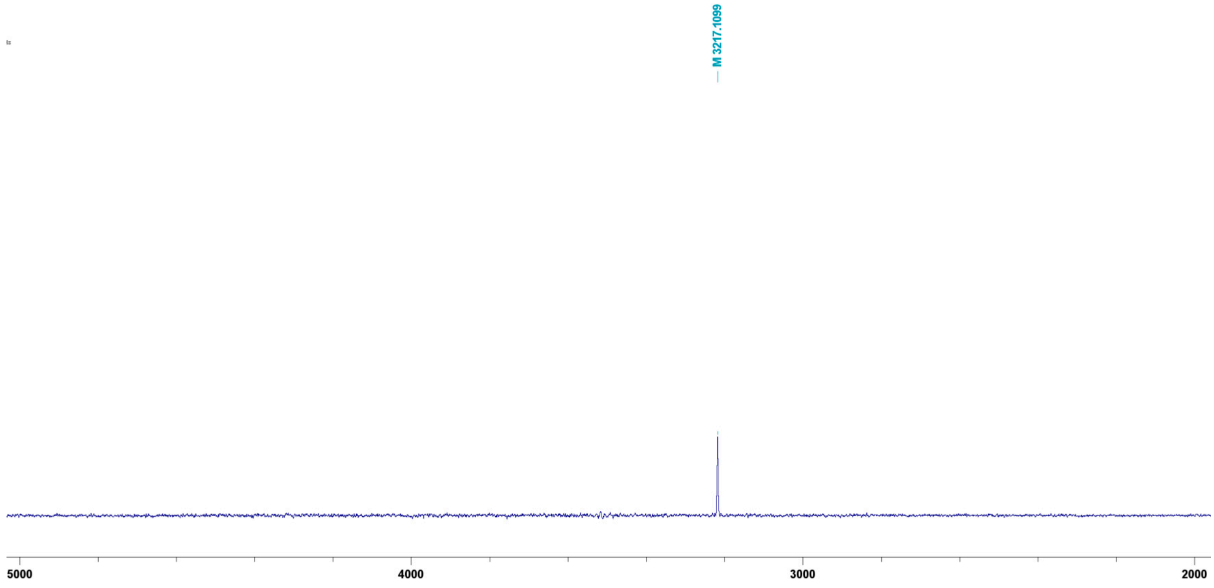

Figure S16. ^{195}Pt NMR spectrum of conjugate **C14** in D_2O.

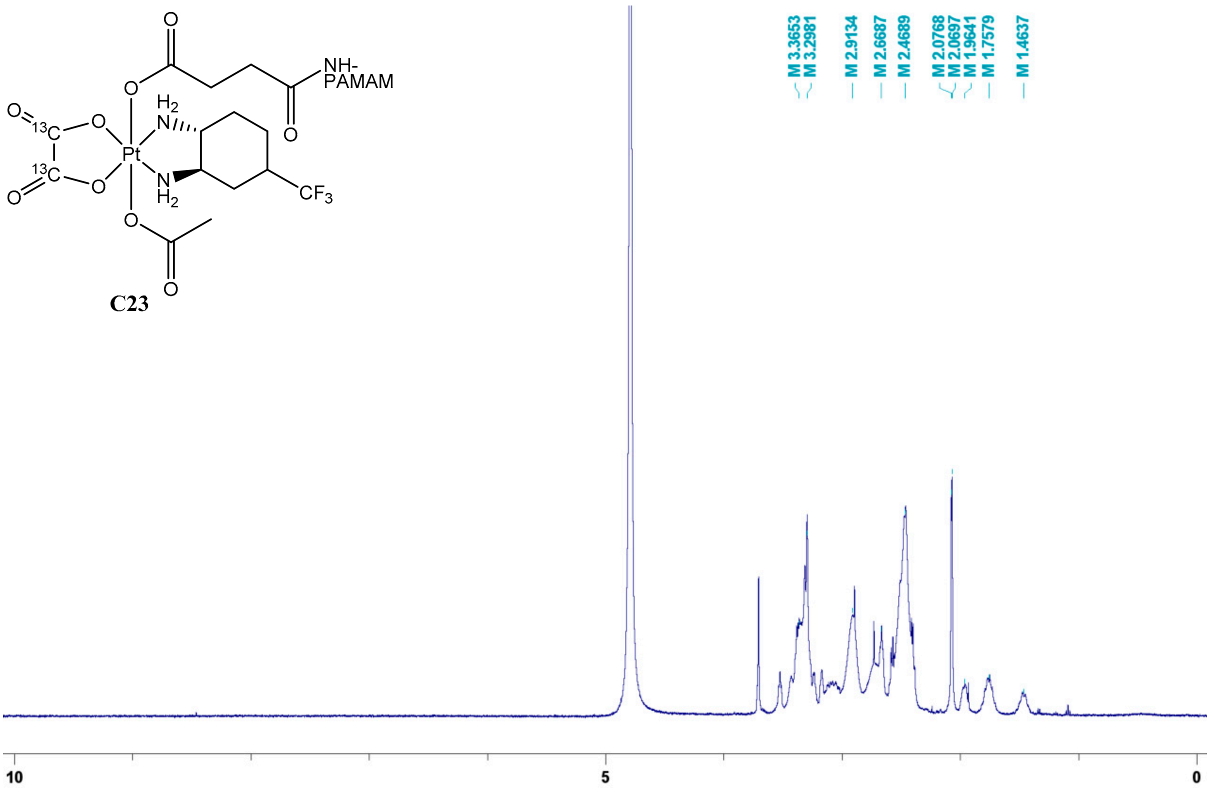

Figure S17. ^{1}H NMR spectrum of conjugate **C23** in D_2O.

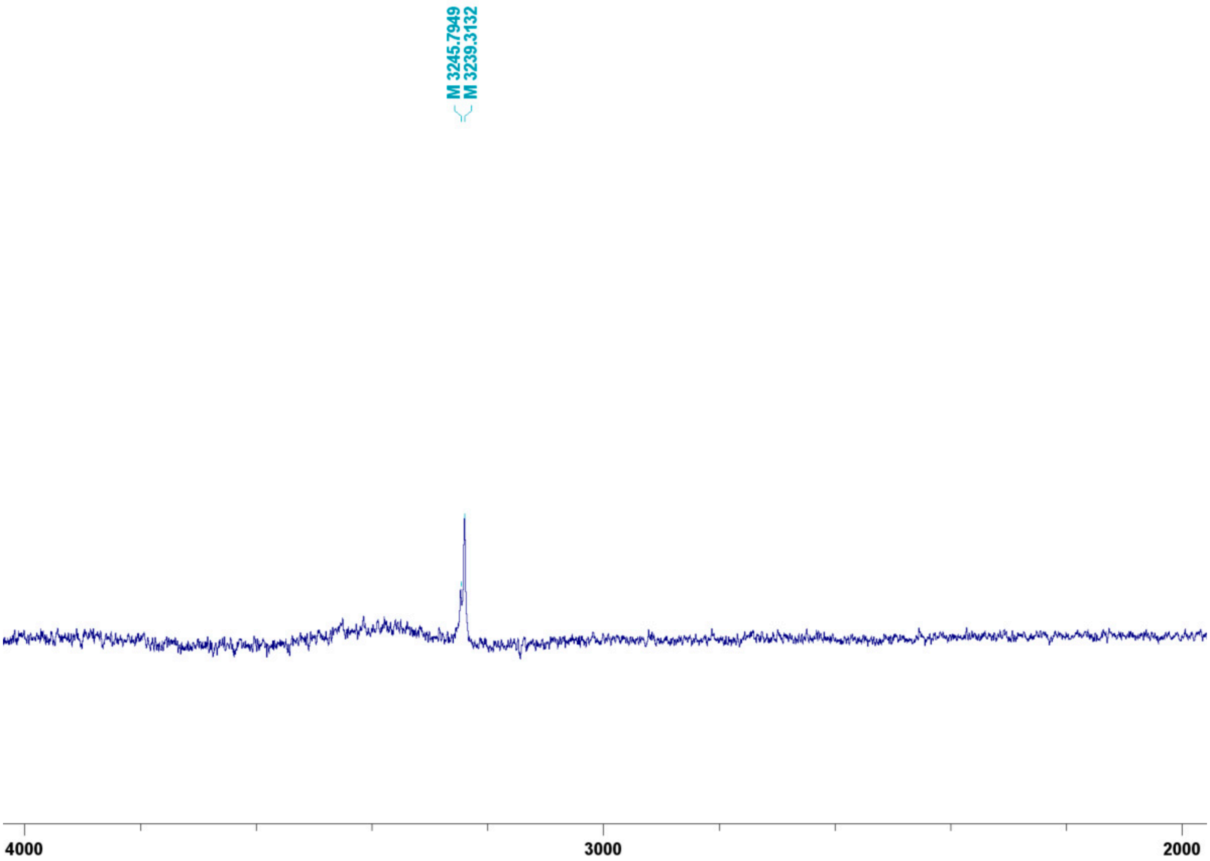

Figure S18. ^{195}Pt NMR spectrum of conjugate **C23** in D_2O.

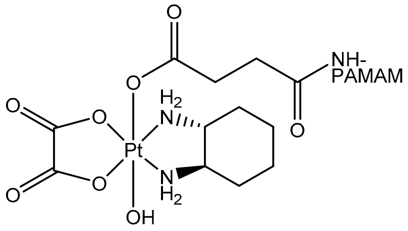

C24

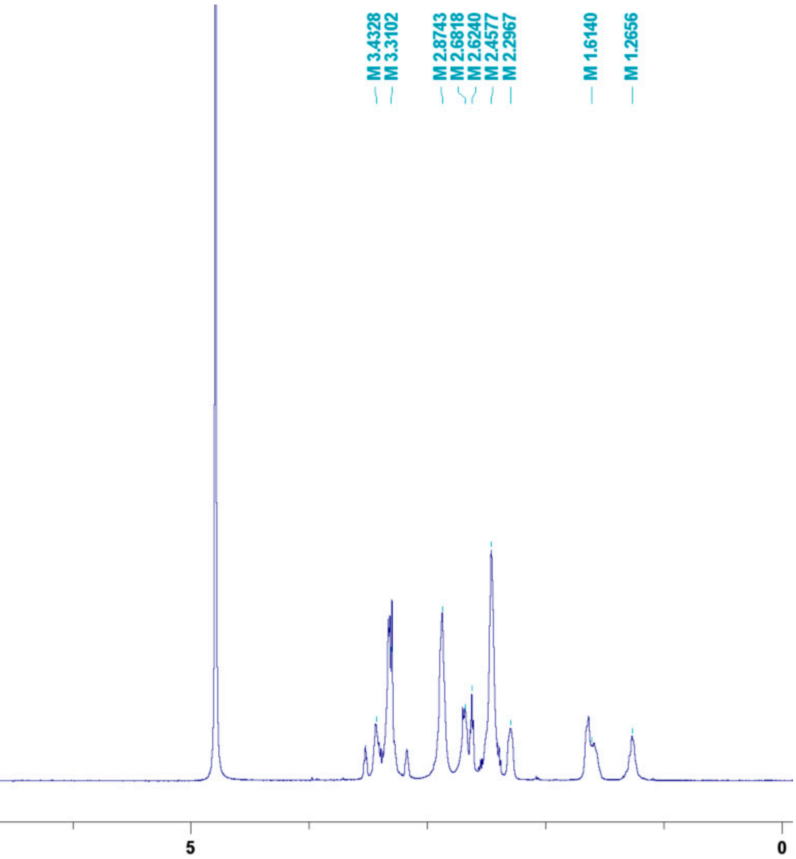

Figure S19. ^{1}H NMR spectrum of conjugate **C24** in D_2O.

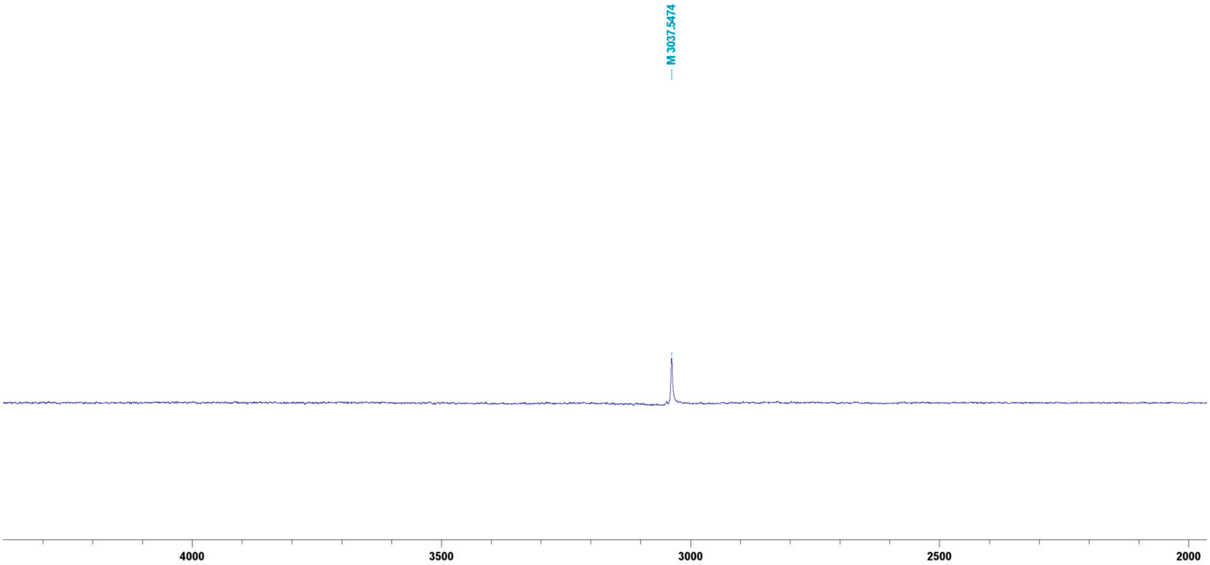

Figure S20. ^{195}Pt NMR spectrum of conjugate **C24** in D_2O.

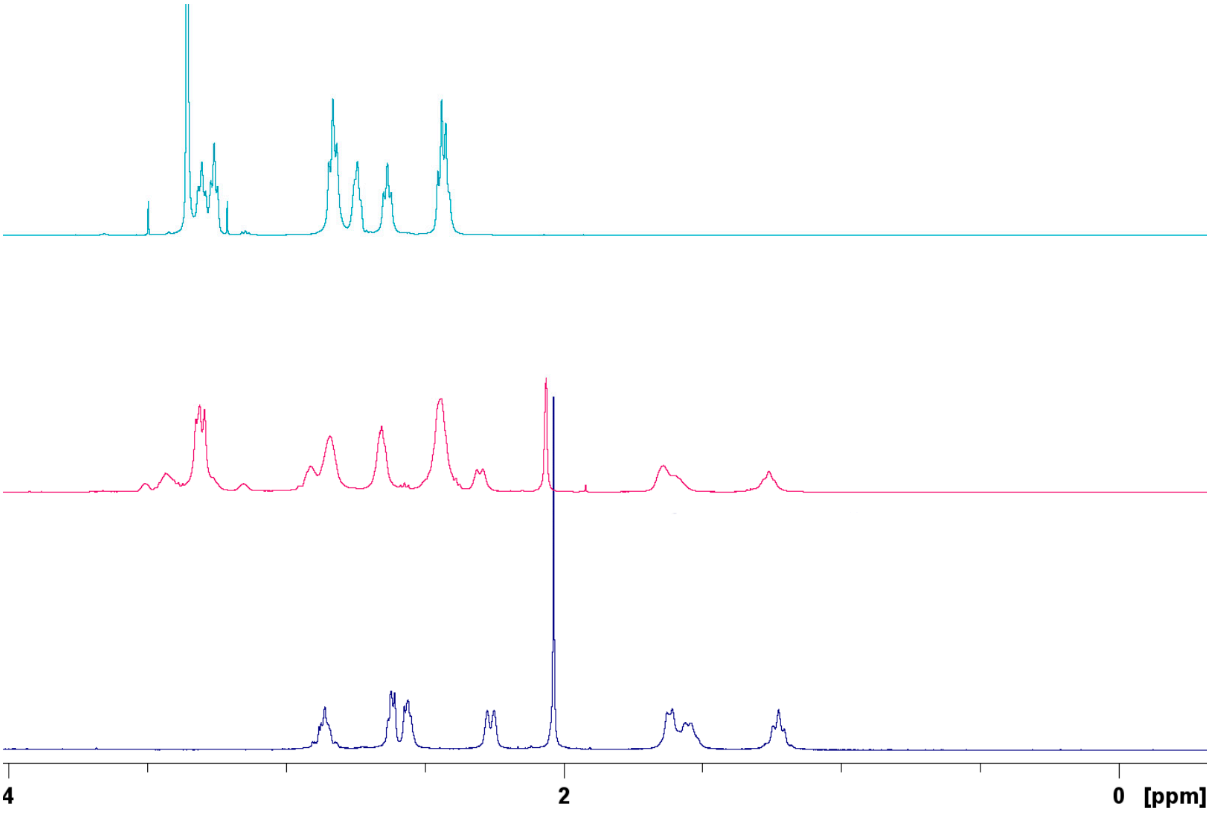

Figure S21. ^{1}H NMR spectra measured in D_2O of G4 PAMAM (top, turquoise), conjugate **C14** (middle, pink) and platinum(IV) complex **3** (bottom, dark blue).

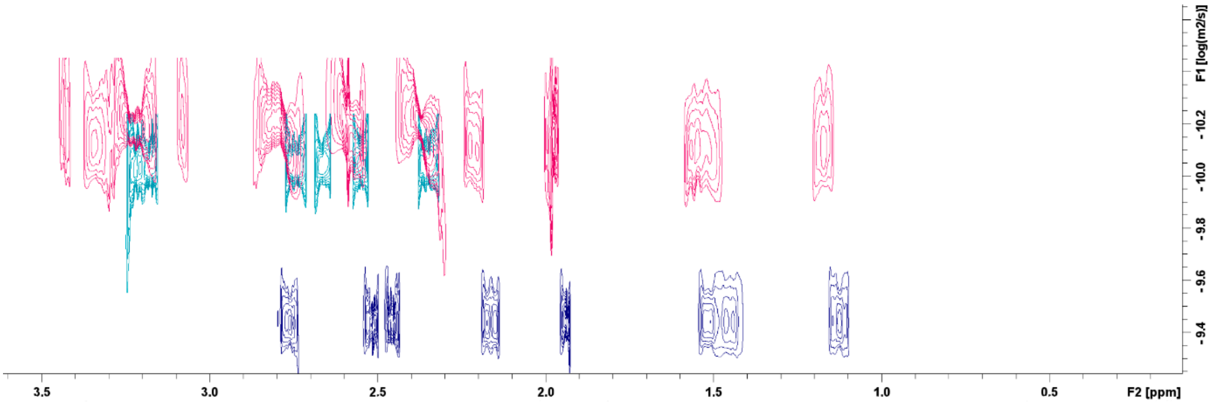

Figure S22. Overlay of DOSY spectra of conjugate **C14** (top, pink), G4 PAMAM (middle, turquoise) and platinum(IV) complex **3** (bottom, dark blue).

3. X-Ray Diffraction Analysis

X-ray intensity data was measured on Bruker D8 Venture diffractometer equipped with multilayer monochromator, Mo K/α INCOATEC micro focus sealed tube and Oxford cooling system. The structure was solved by Direct Methods. Non-hydrogen atoms were refined with anisotropic displacement parameters. Hydrogen atoms were inserted at calculated positions and refined with riding model. The following software was used: Bruker SAINT software package [1] using a narrow-frame algorithm for frame integration, SADABS [2] for absorption correction, OLEX2 [3] for structure solution, refinement, molecular diagrams and graphical user-interface, ShelXle [4] for refinement and graphical user-interface SHELXS-2015 [5] for structure solution, SHELXL-2015 [6] for refinement, Platon [7] for symmetry check. Crystallographic data have been deposited with the Cambridge Crystallographic Data Center with No. CSD 2252655. Copies of data can be obtained free of charge (available online: https://www.ccdc.cam.ac.uk/structures/).

Table S1. Overview of the sample and crystal data, data collection and structure refinement of platinum(IV) complex **3**.

**Identification code**	mo_KrYv410_P212121
**Empirical formula**	$C_{14}H_{26}N_2O_{12}Pt$
**Formula weight**	609.46
**Temperature/K**	100.0
**Crystal system**	orthorhombic
**Space group**	$P2_12_12_1$
**a/Å**	9.1152(2)
**b/Å**	11.2755(2)
**c/Å**	19.5809(4)
**α/°**	90
**β/°**	90
**γ/°**	90
**Volume/Å^3**	2012.49(7)
**Z**	4
**ρ_{calc}g/cm^3**	2.011
**μ/mm^{-1}**	7.037
**F(000)**	1192.0
**Crystal size/mm^3**	0.1 × 0.05 × 0.03
**Radiation**	MoKα (λ = 0.71073)
**2Θ range for data collection/°**	4.16 to 60.364
**Index ranges**	-12 ≤ h ≤ 12, -15 ≤ k ≤ 15, -27 ≤ l ≤ 23
**Reflections collected**	21714
**Independent reflections**	5890 [R_{int} = 0.0657, R_{sigma} = 0.0684]
**Data/restraints/parameters**	5890/12/280
**Goodness-of-fit on F^2**	1.023
**Final R indexes [I>=2σ (I)]**	R_1 = 0.0346, wR_2 = 0.0485
**Final R indexes [all data]**	R_1 = 0.0484, wR_2 = 0.0518
**Largest diff. peak/hole / e Å^{-3}**	0.85/-1.16
**Flack parameter**	-0.025(6)

Table S2. Overview of bond lengths of platinum(IV) complex **3**.

Atom	Atom	Length [Å]	Atom	Atom	Length [Å]

Pt1	O1	2.004(4)	N1	C1	1.490(8)
Pt1	O5	2.010(4)	N2	C2	1.499(8)
Pt1	O6	2.009(4)	C1	C2	1.489(9)
Pt1	O9	2.002(4)	C1	C6	1.531(9)
Pt1	N1	2.031(5)	C2	C3	1.539(9)
Pt1	N2	2.034(5)	C3	C4	1.530(9)
O1	C7	1.308(7)	C4	C5	1.510(10)
O2	C7	1.237(7)	C5	C6	1.527(9)
O3	C10	1.194(7)	C7	C8	1.508(8)
O5	C11	1.313(8)	C8	C9	1.510(8)
O6	C12	1.304(7)	C9	C10	1.498(9)
O7	C11	1.210(8)	C10	O4	1.353(14)
O8	C12	1.217(8)	C10	O4Z	1.362(13)
O9	C13	1.301(8)	C11	C12	1.543(9)
O10	C13	1.227(8)	C13	C14	1.494(9)

Table S3. Overview of angles of platinum(IV) complex **3**.

Atom	Atom	Atom	Angle [°]	Atom	Atom	Atom	Angle [°]
O1	Pt1	O5	95.13(18)	C1	C2	N2	106.3(5)
O1	Pt1	O6	94.81(18)	C1	C2	C3	112.0(5)
O1	Pt1	N1	84.0(2)	C4	C3	C2	108.9(6)
O1	Pt1	N2	86.5(2)	C5	C4	C3	111.8(6)
O5	Pt1	N1	97.0(2)	C4	C5	C6	111.1(6)
O5	Pt1	N2	178.3(2)	O1	C7	C8	113.6(5)
O6	Pt1	O5	84.74(16)	O2	C7	O1	125.0(5)
O6	Pt1	N1	177.9(2)	O2	C7	C8	121.4(5)
O6	Pt1	N2	94.7(2)	C7	C8	C9	114.8(5)
O9	Pt1	O1	177.1(2)	C10	C9	C8	115.8(5)
O9	Pt1	O5	86.66(19)	O3	C10	C9	124.4(6)
O9	Pt1	O6	83.11(18)	O3	C10	O4	119.6(8)
O9	Pt1	N1	98.1(2)	O3	C10	O4Z	121.5(8)
O9	Pt1	N2	91.7(2)	O4	C10	C9	112.1(8)
N1	Pt1	N2	83.59(19)	O4Z	C10	C9	111.4(7)
C7	O1	Pt1	124.4(4)	O5	C11	C12	116.2(7)
C11	O5	Pt1	111.2(4)	O7	C11	O5	123.8(7)
C12	O6	Pt1	111.2(4)	O7	C11	C12	120.0(7)
C13	O9	Pt1	123.3(5)	O6	C12	C11	116.7(7)
C1	N1	Pt1	108.8(4)	O8	C12	O6	122.1(7)
C2	N2	Pt1	108.4(4)	O8	C12	C11	121.2(7)
N1	C1	C6	113.1(6)	O9	C13	C14	112.4(7)
C2	C1	N1	108.2(5)	O10	C13	O9	125.4(7)
C2	C1	C6	111.3(6)	O10	C13	C14	122.1(7)
N2	C2	C3	112.3(6)	C5	C6	C1	109.8(6)

4. Concentration-Effect Curves

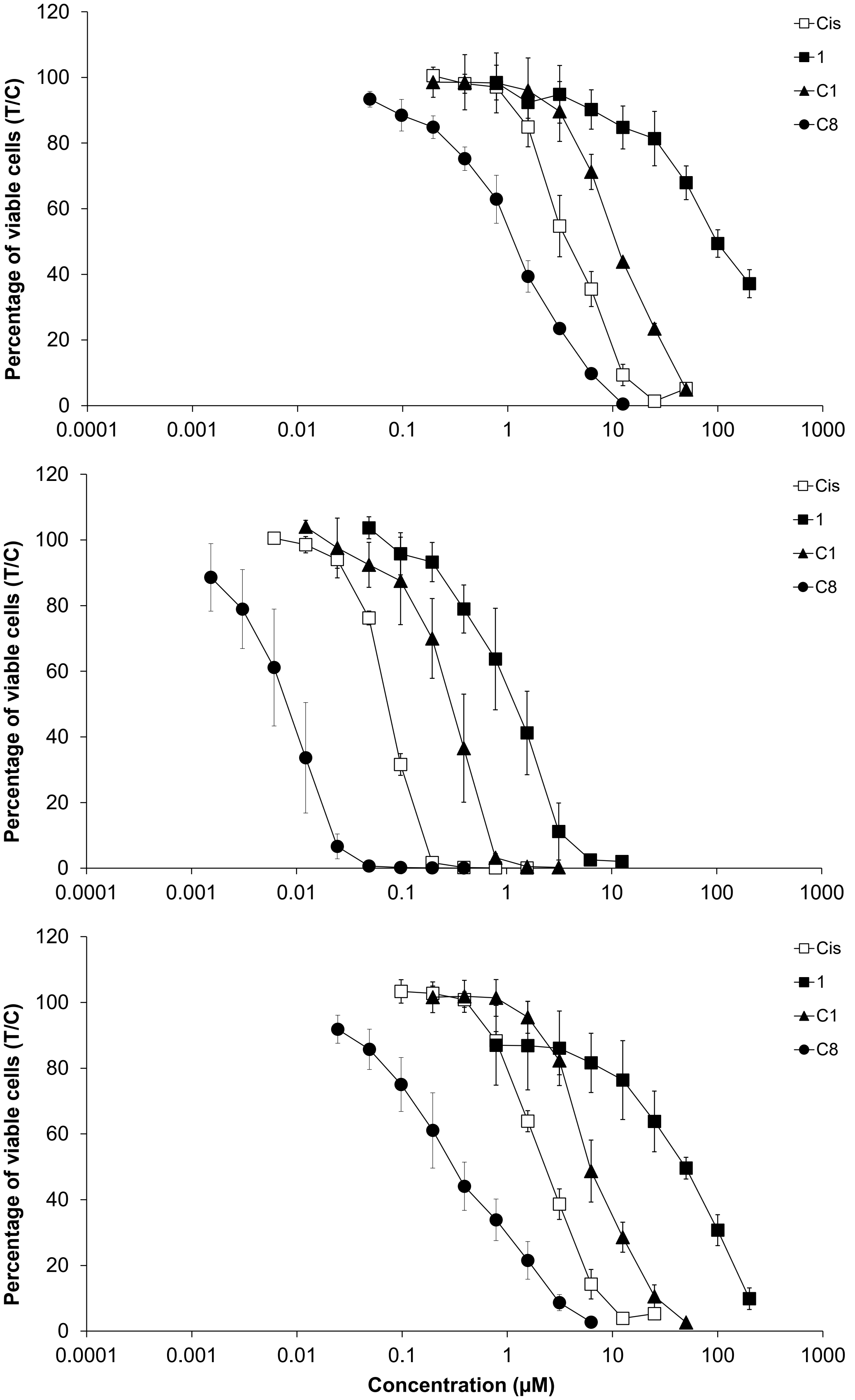

Figure S23. Concentration-effect curves of cisplatin, **1**, **C1** and **C8** in A549 (top), CH1/PA-1 (middle) and SW480 (bottom) cells, obtained by MTT assays with 96 h exposure time. Values are means ± standard deviations from at least three independent experiments.

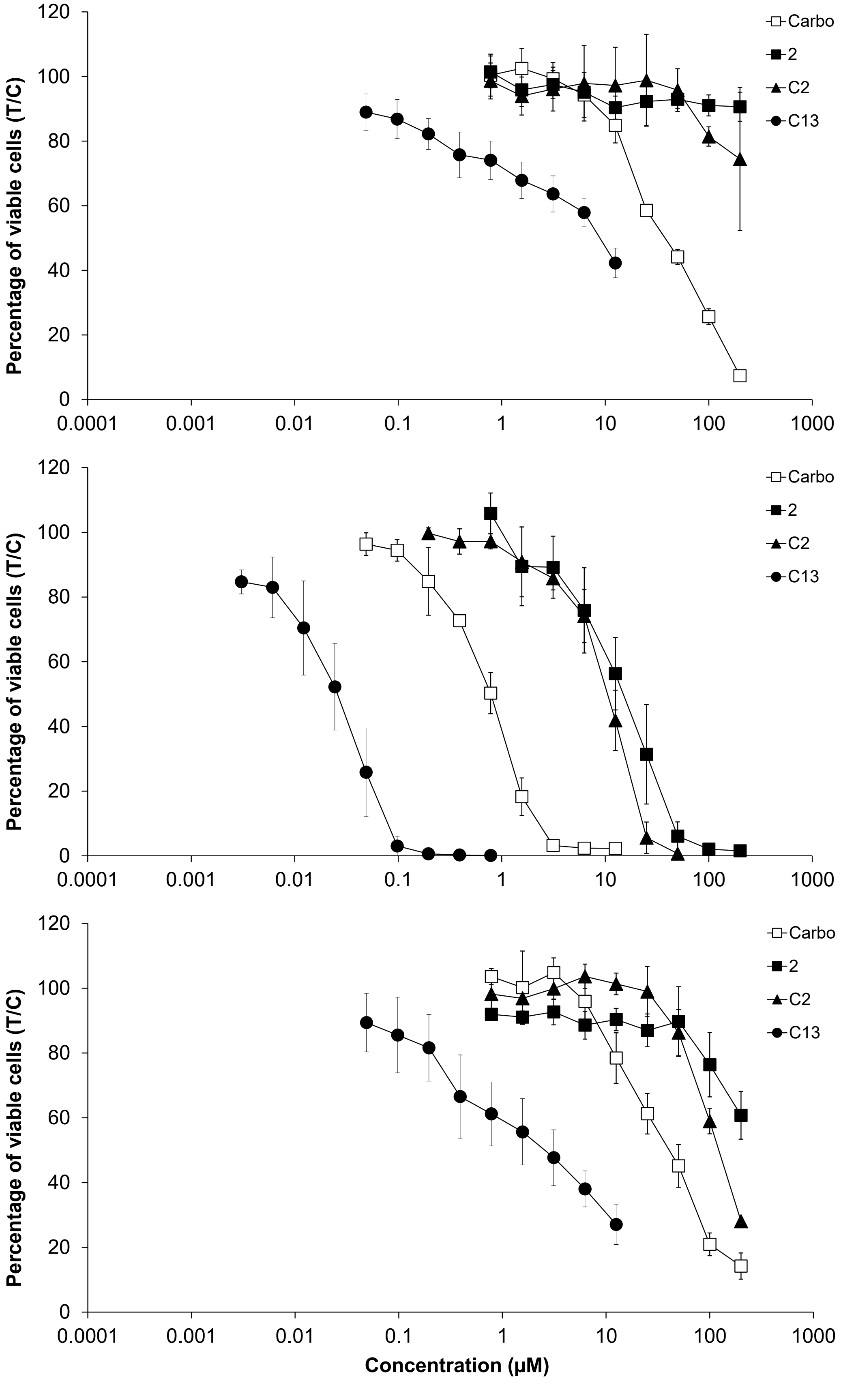

Figure S24. Concentration-effect curves of carboplatin, **2**, **C2** and **C13** in A549 (top), CH1/PA-1 (middle) and SW480 (bottom) cells, obtained by MTT assays with 96 h exposure time. Values are means ± standard deviations from at least three independent experiments.

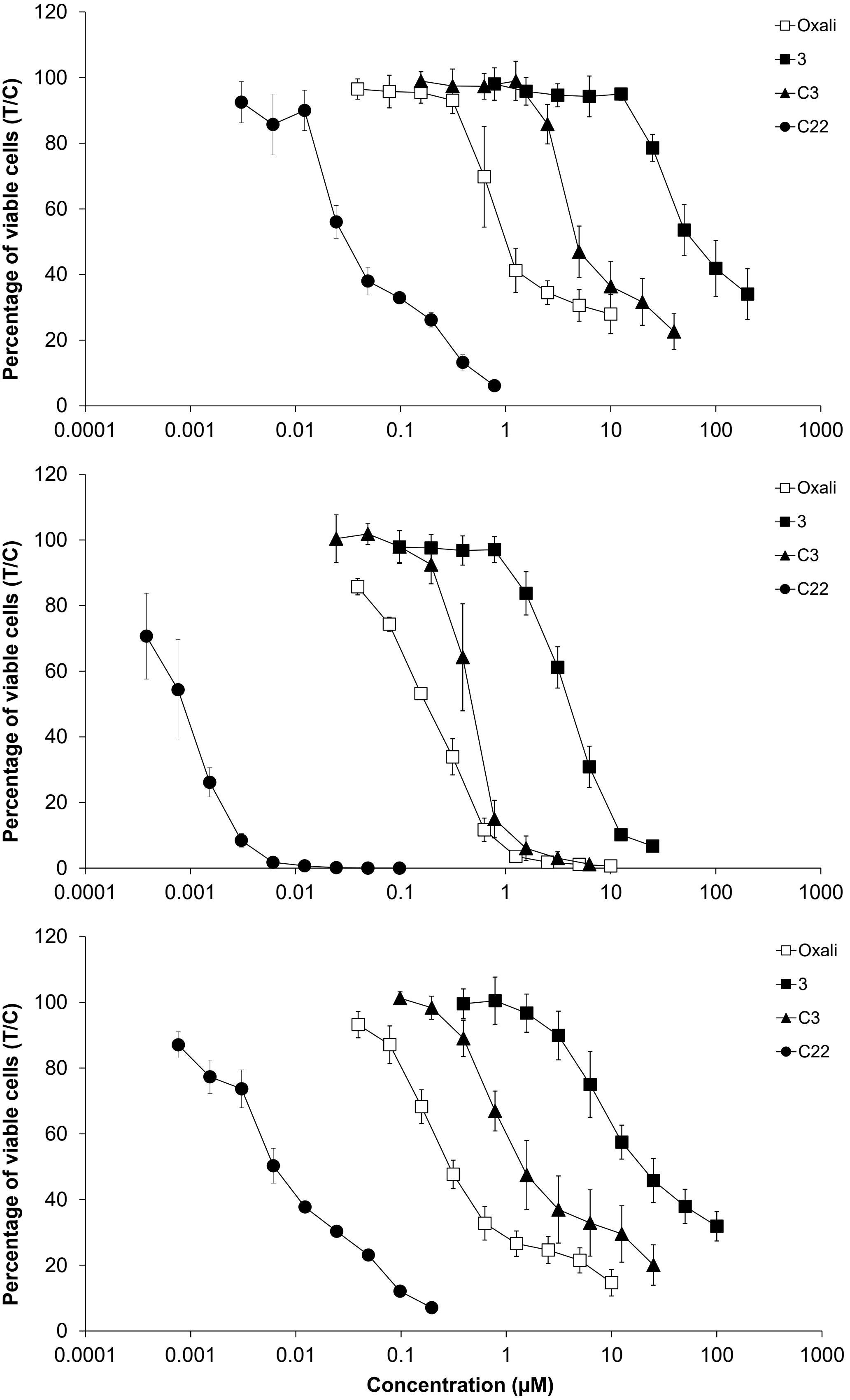

Figure S25. Concentration-effect curves of oxaliplatin, **3**, **C3** and **C22** in A549 (top), CH1/PA-1 (middle) and SW480 (bottom) cells, obtained by MTT assays with 96 h exposure time. Values are means ± standard deviations from at least three independent experiments.

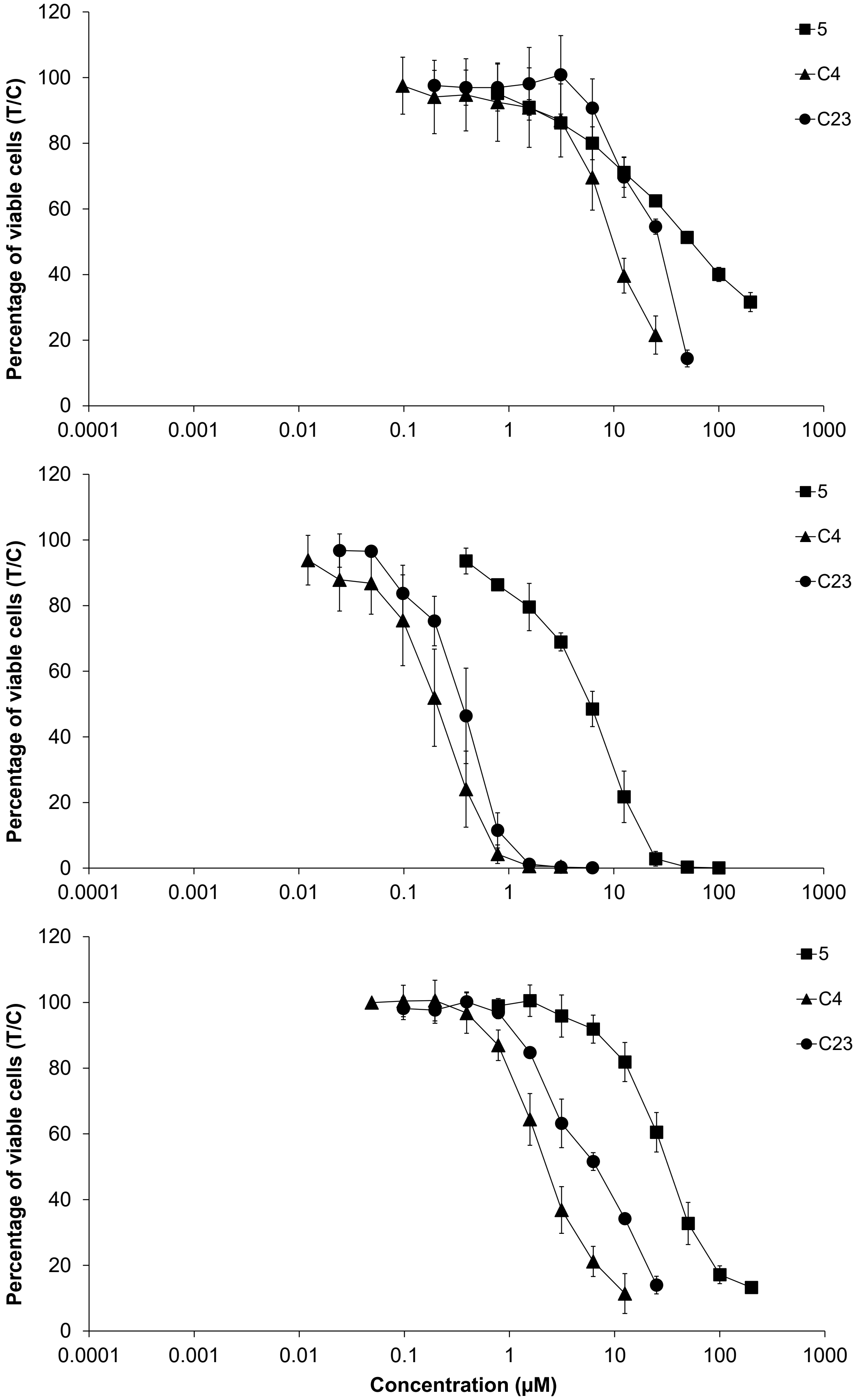

Figure S26. Concentration-effect curves of **5**, **C4** and **C23** in A549 (top), CH1/PA-1 (middle) and SW480 (bottom) cells, obtained by MTT assays with 96 h exposure time. Values are means ± standard deviations from at least three independent experiments.

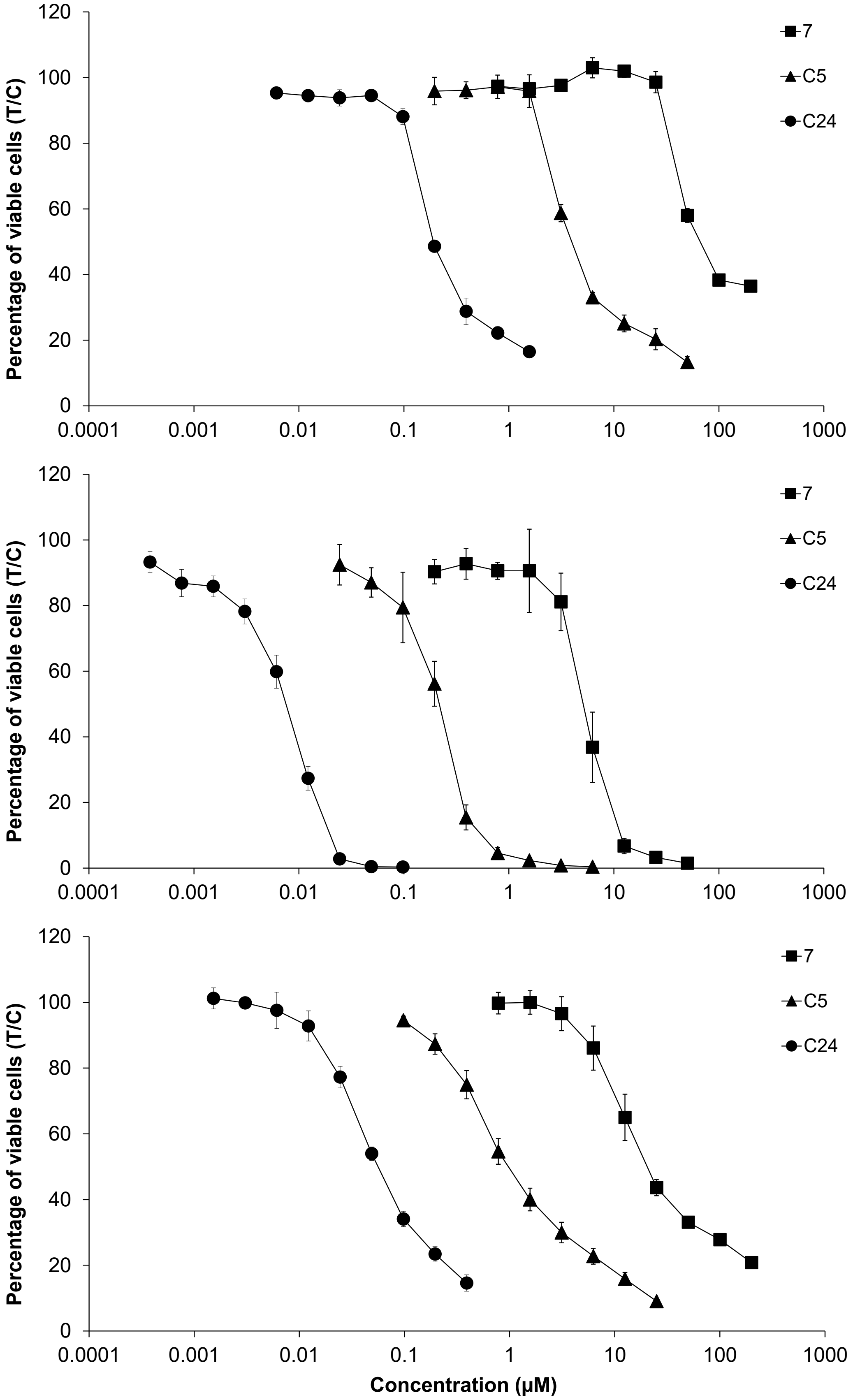

Figure S27. Concentration-effect curves of **7**, **C5** and **C24** in A549 (top), CH1/PA-1 (middle) and SW480 (bottom) cells, obtained by MTT assays with 96 h exposure time. Values are means ± standard deviations from at least three independent experiments.

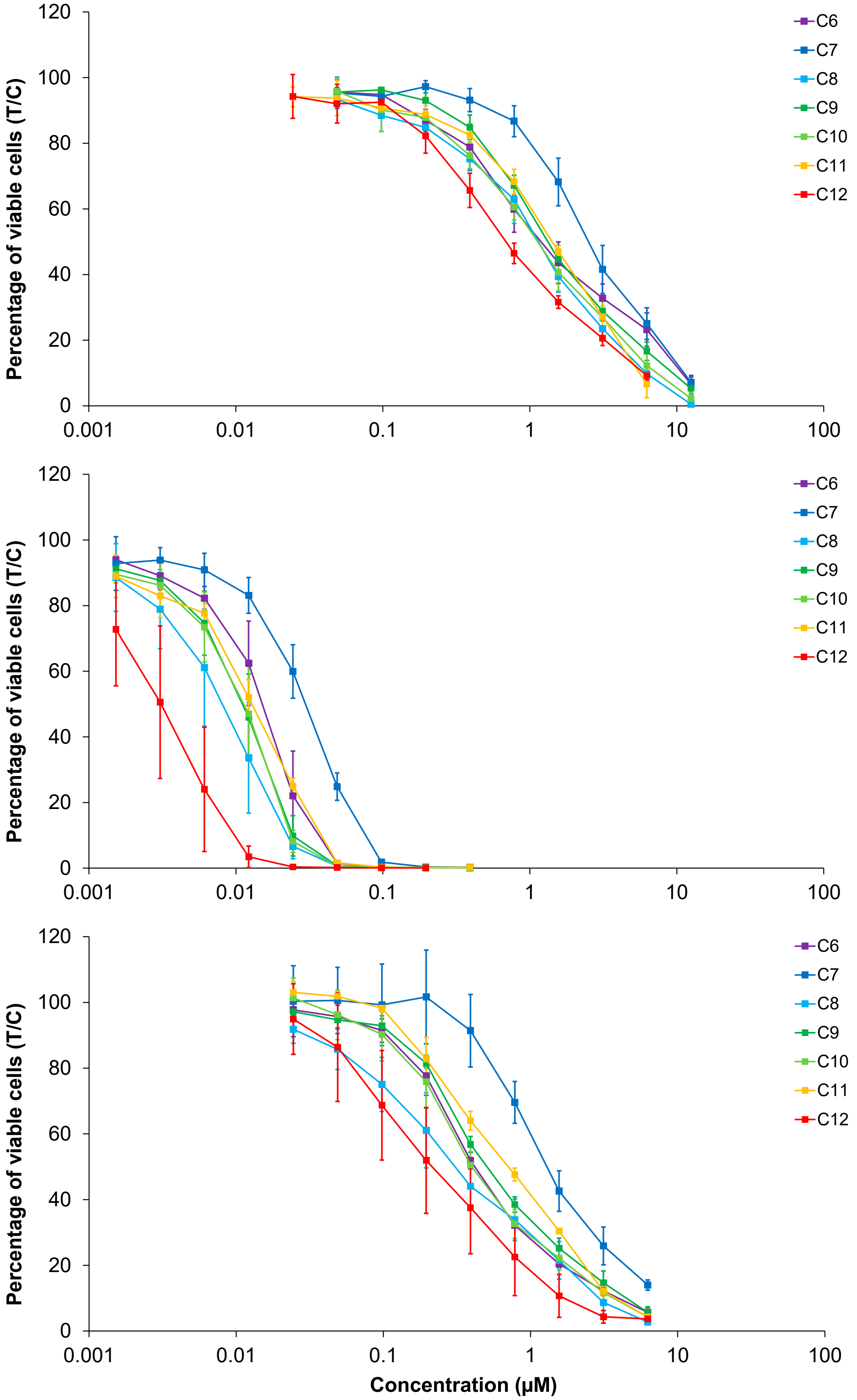

Figure S28. Concentration-effect curves of **C6**–**C12** in A549 (top), CH1/PA-1 (middle) and SW480 (bottom) cells, obtained by MTT assays with 96 h exposure time. Values are means ± standard deviations from at least three independent experiments. Compounds are color-coded according to their Pt loading from purple (lowest) to red (highest).

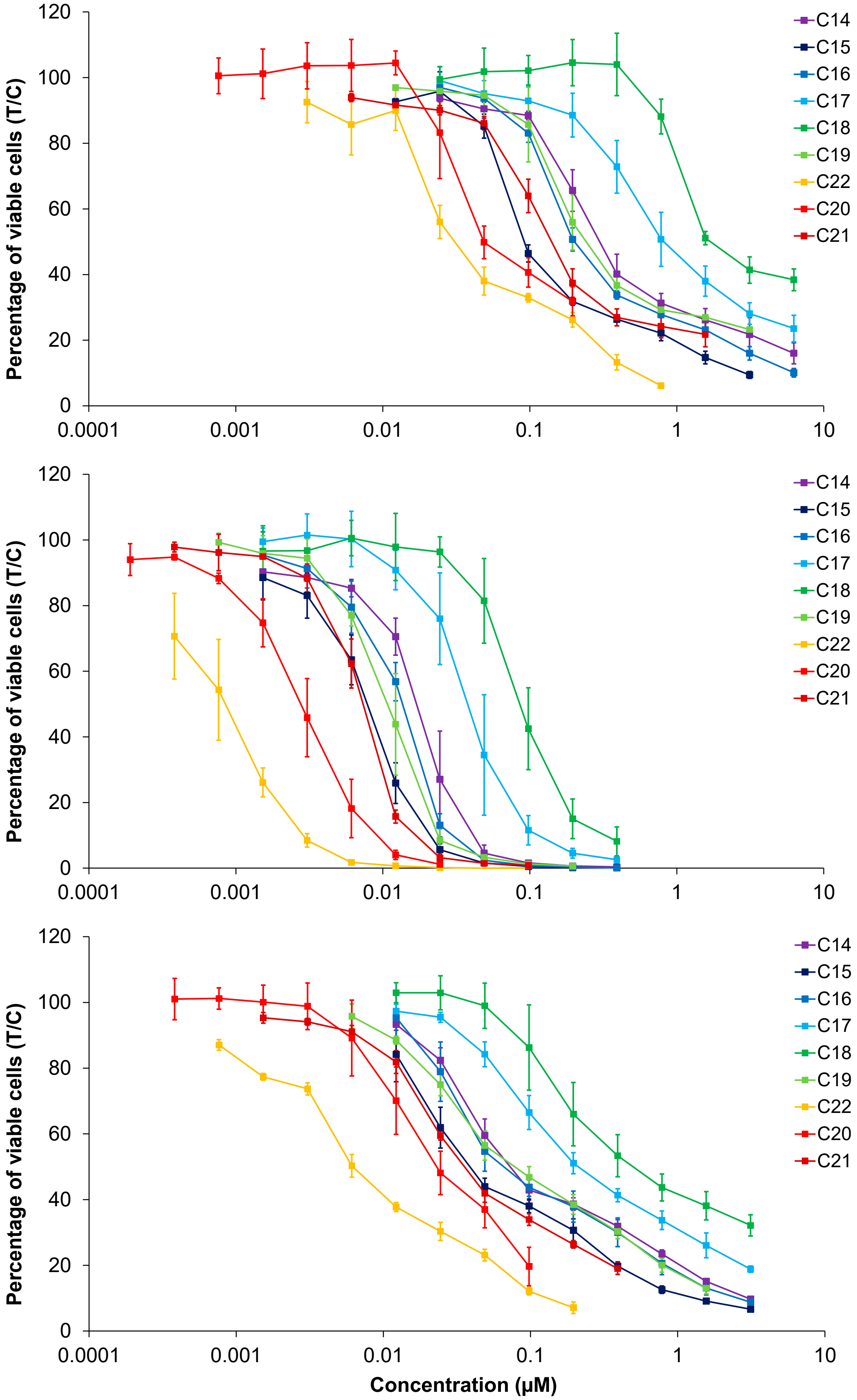

Figure S29. Concentration-effect curves of **C14–C21** in A549 (top), CH1/PA-1 (middle) and SW480 (bottom) cells, obtained by MTT assays with 96 h exposure time. Values are means ± standard deviations from at least three independent experiments. Compounds are color-coded according to their Pt loading from purple (lowest) to red (highest).

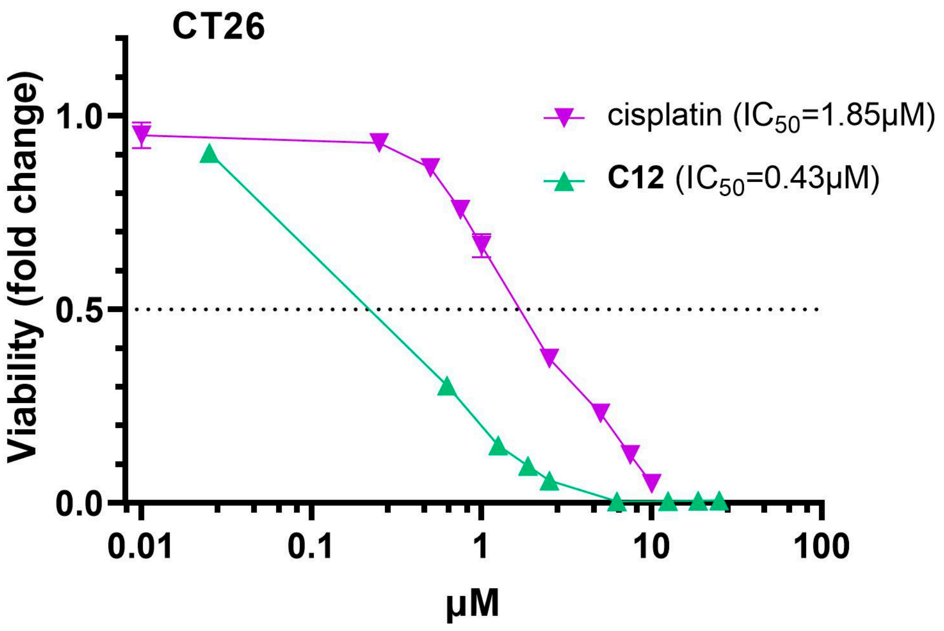

Figure S30. Concentration-effect curves of cisplatin and **C12** at the indicated doses (equimolar concerning cisplatin) in CT26 cells, obtained by MTT assays with 72 h exposure time.

5. In Vivo Data

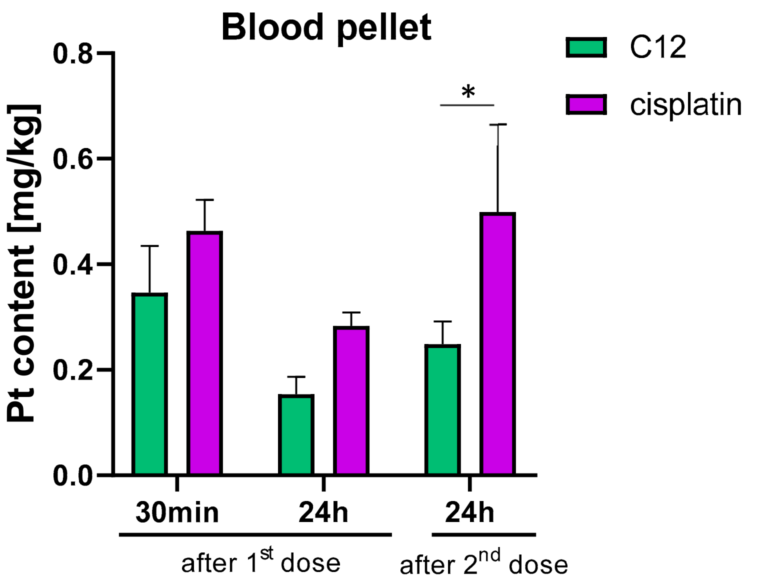

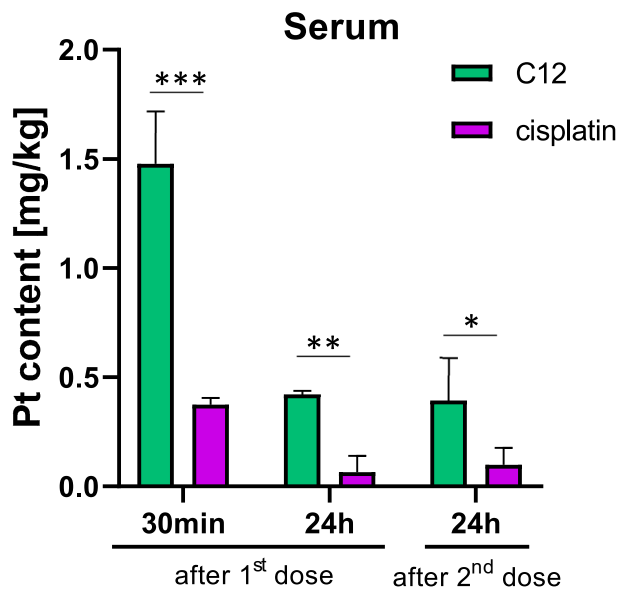

Figure S31. Comparison of platinum amount in the blood pellet and serum of conjugate **C12** and cisplatin. Significances were determined via Log-rank test (LRT) and Gehan-Breslow-Wilcoxon test (GBWT) with following abbreviations: ns = not significant, * $p < 0.05$, ** $p < 0.01$, *** $p < 0.001$, **** $p < 0.0001$.

6. References

1. Bruker SAINT v8.38B Copyright © 2005-2019 Bruker AXS.
2. Sheldrick, G. M. (1996). SADABS. University of Göttingen, Germany.
3. Dolomanov, O. V.; Bourhis, L.J.; Gildea, R.J.; Howard, J.A.K.; Puschmann, H. OLEX2: A Complete Structure Solution, Refinement and Analysis Program. *J Appl Crystallogr* **2009**, *42*, 339–341, doi:10.1107/S0021889808042726.
4. Hübschle, C.B.; Sheldrick, G.M.; Dittrich, B. ShelXle: A Qt Graphical User Interface for SHELXL. *J Appl Crystallogr* **2011**, *44*, 1281–1284, doi:10.1107/S0021889811043202.
5. Sheldrick, G. M. (2015). SHELXS v 2016/4 University of Göttingen, Germany.
6. Sheldrick, G. M. (2015). SHELXL v 2016/4 University of Göttingen, Germany.
7. Spek, A.L. Structure Validation in Chemical Crystallography. *Acta Crystallogr D Biol Crystallogr* **2009**, *65*, 148–155, doi:10.1107/S090744490804362X.
